# Problems associated with the ATC system of drug classification

**DOI:** 10.1007/s00210-025-04833-1

**Published:** 2025-12-01

**Authors:** Lilly Josephine Bindel, Roland Seifert

**Affiliations:** Institute of Pharmacology, Hannover Medical School, Carl-Neuberg-Str. 1, 30625 Hannover, Germany

**Keywords:** ATC, Classification, Pharmacological nomenclature, Drug utilization research, Innovative medicines

## Abstract

The Anatomical Therapeutic Chemical (ATC) classification system is the international standard for drug utilisation studies. However, structural and conceptual issues remain inadequately addressed. This analysis evaluates the ATC system with respect to consistency, completeness and terminology to identify systemic weaknesses and point out potential alternatives. A systematic analysis of all 14 ATC main groups and their sublevels was conducted based on the “ATC/DDD Index 2025” and the official WHO “2025 guideline”. The classification logic, coverage of therapeutic areas, handling of combination products, and terminology were examined. Discrepancies were recorded within groups and across the system as a whole. Widespread inconsistencies became apparent. Classification principles (anatomical, therapeutic, pharmacological, chemical, miscellaneous) are mixed within and across levels, causing structural incoherence. Frequent use of "X/ miscellaneous" categories highlights inadequacies in the classification logic, along with inconsistent handling of combination preparations. Drugs with multiple indications are fragmented across groups, with frequent overlaps and duplications. Terminology is often vague or outdated, obscuring pharmacological mechanisms and reflecting a historical rather than scientific rationale. Overall, the ATC system is characterised by systematic deficiencies rather than isolated irregularities. The ATC classification no longer adequately represents modern pharmacotherapy. Its reliance on single-indication logic, miscellaneous categories and outdated terminology distorts drug utilisation analyses and limits its applicability in clinical and research contexts. A mechanistically oriented system based on pharmacological properties and molecular targets would provide a more consistent, transparent and adaptable framework that is better suited to contemporary drug development, multi-indicational use and rational prescribing.

**Supplementary Information:**

The online version contains supplementary material available at 10.1007/s00210-025-04833-1.

## Introduction

**Fig. 1:**
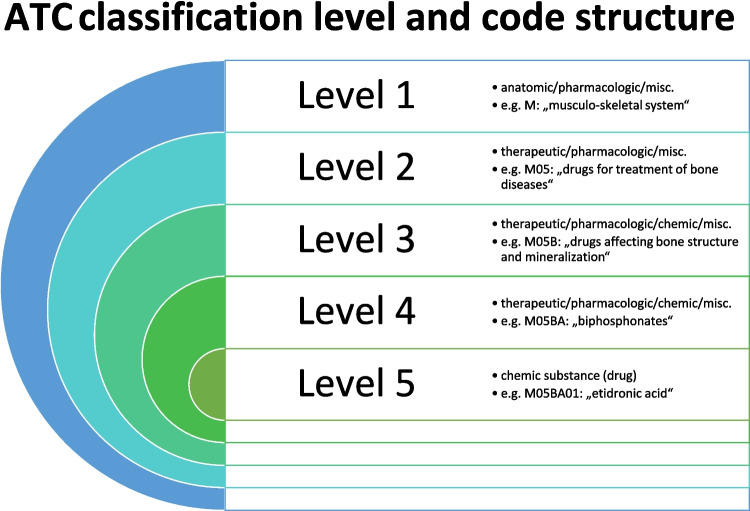
Overview of the ATC classification levels and code structure. For each level (1–5), the possible classification criteria are presented, along with an example

The Anatomical Therapeutic Chemical (ATC) classification is a system for categorizing therapeutic drugs, structured into 14 main groups and 5 levels, with a disease-oriented focus (Seifert and Alexander 2022) (Table 1, Fig. 1). It was first introduced in the 1960 s, and developed by the drug utilization research group (DURG) (WHO 2025a). In 1980, the World Health Organization (WHO) recommended the ATC system as the “state of the art” as the international standard for drug utilization studies, followed by the establishment of the “WHO Collaborating Centre for Drug Statistics Methodology” in 1982 (WHO 2018). A globalization of the system was undertaken in 1996 to enable international drug consumption analyses, particularly in developing countries (WHO 2018). The availability of a comprehensive drug classification system is essential, incorporating FAIR data principles for international drug utilisation research to improve clinical practice (Wilkinson et al. 2016; Salgado-Baez et al. 2025).

**Table 1:** Fundamental principles of the ATC classification structure. For each aspect, the corresponding ATC principles are presented, along with an explanation and an example

Aspect	ATC principle (WHO 2022)	explanation	example (WHO^2024^)
Number of ATC codes	one ATC code per pharmaceutical substance	often more than one indication; prioritized to assign based on mechanism of action instead of therapy; one ATC group can include drugs with different indications	any drug, e.g. amoxicillin (J01CA04)
Number of ATC codes	only one ATC code for each route of administration	classification of drug by active ingredients, route of administration and strength	only one ATC code for topical use and systemic use
Number of ATC codes	more than one ATC code for a medicinal substance; based on different routes of administration or strengths	only if difference is due to different therapeutic use	prednisolone
Number of ATC codes	one ATC code for different pharmacokinetics in the same active ingredient	same ATC code for immediate and slow-release tablets	metoprolol
Number of ATC codes	different ATC codes for stereoisomers and prodrugs	consideration of different characteristics and indications	pivmecillinam (J01AC08), mecillinam (J01CA11)
Nomenclature	preference of international non-proprietary names (INN), alternative British approved name (BAN) or United States adopted name (USAN)	use of name for pharmaceutical substance, not brand name	acetylsalicylic acid (ASS) instead "Aspirin"
New ATC groups	new specific 4th levels only induced when at least two substances with marketing authorisations fit in the group, as well as a benefit for utilization analysis has to be created	priority to avoid specified groups with only one substance, while X groups can consist of only one drug	
Inclusion criteria	new entries on requests; application for marketing authorisation for drug; availability of INN name	drug has to be approved for use, needs an official name, listing has to be requested	-
Classification	avoidance of only one substance per subgroup	mainly based on therapeutic or pharmacological classes, with rather broad groups	C cardiovascular system—> C08 calcium channel blockers (main indication can be coronary heart disease or hypertension)
Classification	combination products only included once	classification based on main therapeutic use; sometimes separate combination categories	antiparasitics + antibacterials antibacterials for systemic use(J01R), since effect against bacteria is considered the most important in this combination
Assignation of indication	only one main indication per drug, discussion and decision about issues in the "WHO International Working Group for Drug Statistics Methodology"	a drug can have more than one equally important indications	hormones
"Other" groups (X)	new drug not clearly belonging to any existing ATC 4th level is placed in an X group ("other" group) in the relevant 3rd level	new and innovative pharmaceutical substances often classified in an X group	levetiracetam (N03AX)

In previous studies by our group, the ATC system was applied for drug utilization research (Bindel and Seifert 2025a, 2025b, 2025c). During this work, several limitations and sources of confusion inherent in the ATC system became apparent. These include incomplete coverage of therapeutic areas with relevant drug classes missing, substances listed in several main groups, and allocations of drug classes that appear inconsistent or illogical. Such issues are particularly evident in the area of nervous system drugs (Sathyanarayana and Andrade 2016). They lead to restrictions in drug consumption analyses, including distortions due to excluded substances, as well as large “miscellaneous/X” groups that encompass a substantial share of substances but lack logic and structure (Ludwig et al. 2025; Filippo Caraci et al. 2017).

This study aims to conduct a comprehensive analysis of the ATC system with regard to its drug classification, structural organisation and nomenclature. We aim to determine whether the discrepancies identified are confined to specific groups or whether they represent cross-group, systematic problems that point to limitations in the classification itself. By doing so, we seek to contribute to the development of a classification framework that is more precise, consistent and comprehensive, alongside an improved, rational nomenclature. Such changes would increase the granularity and quality of findings in drug utilisation research, while also improving orientation and supporting rational drug use in clinical practice.

## Methods

### Data collection

This study is based on the “ATC/DDD Index 2025” of the WHO (WHO 2024). This index, available in a searchable online version (https://atcddd.fhi.no/atc_ddd_index/), was used together with the official “2025 guideline” (https://atcddd.fhi.no/filearchive/publications/2025_guidelines__final_web.pdf) (WHO 2025b).

### Classification analysis

All 14 main ATC groups (A, B, C, D, G, H, J, L, M, N, P, R, S and V) and their sublevels were systematically analysed. The classification structure of each group was examined to identify potential discrepancies. Individual country modifications, such as for Germany (Günther et al. 2025), were compared with the international ATC system. The defined daily dose (DDD) measure was not analysed.

**Fig. 2:**
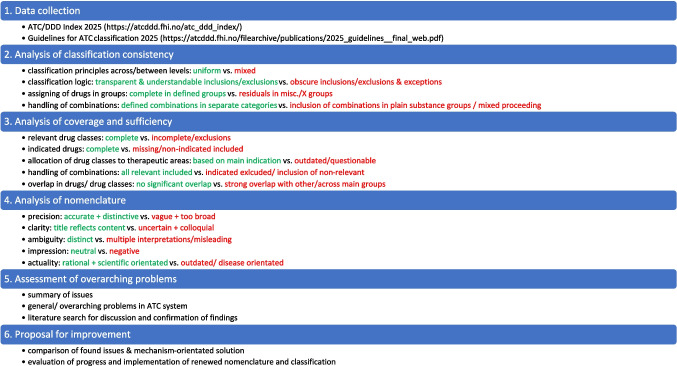
Methodological approach and algorithm for evaluating appropriate versus problematic characteristics for the ATC classification system

The evaluation focused on three dimensions: consistency (uniformity of classification logic), sufficiency (completeness of therapeutic areas and drug groups), and nomenclature (precision and rationality of terminology). These results were then evaluated to identify overarching issues across the system. The applied criteria and the methodological approach are summarized in Fig. 2.

### Proposal for improvement

Building on the critique of the ATC system, a mechanism-based classification and nomenclature was introduced. This approach was then compared with the current ATC framework, with the advantages outlined and the progress of its implementation evaluated.

## Results

### A – alimentary tract and metabolism

This group covers the gastrointestinal tract and metabolic processes, structured by diverse aspects such as anatomy, therapeutic area, pharmacological effect, and chemical structure. Its classification is partly insufficient, as indicated by the miscellaneous category “other” (A16).

Overlaps occur extensively with other main groups, e.g. “systemic antiinfectives” (J) and “cardiovascular system” (C) (Table 2 and S1). This results in the exclusion of drugs like vitamin B12 and vitamin K, which are instead found in “blood and blood forming organs” (B03BA, B02BA). This also occurs across subgroups, for example “stomatological preparations” (A01) overlapping with “intestinal antiinfectives” (A07), or “vitamins” (A11). Also, this results in incomplete subgroups, for example by the exclusion of semaglutide of “antiobesity” (A07), but listing in “drugs used in diabetes” (A10B) instead. Handling of combinations is inconsistent, e.g. in “drugs for acid-related disorders” (A02), where combinations in “GORD” (A02B) include substances from “antacids” (A02A).

**Table 2:** Summary of ATC main groups with regard to the most prominent problems in terms of consistency, sufficiency, and nomenclature. A comprehensive evaluation for all subgroups can be found in Table S1

Main group	Consistency (inconsistencies and overlaps)	Sufficiency (missing drugs/classes)	Nomenclature issues (problematic terms)	Sources
A – Alimentary tract and metabolism	Mixed therapeutic/chemical grouping; overlaps with D (corticosteroids), R (throat/mouth), V (diet products), and between subgroups	vitamins (A11), parenteral minerals (A12), colecalciferol, sodium fluoride	“anti-” (antidiarrheals, antiobesity), “therapy” (bile and liver therapy), “other” (A16)	Addy and Fugit 1989; Savarino et al. 2017, 2021; Drossman et al. 2018; Singh et al. 2022; Herrstedt 2018; Athavale et al. 2020; Fonte et al. 2015; Ali et al. 2023; Gilgenkrantz et al. 2025; Luthra et al. 2019; Rao and Brenner 2021; Lee 2015; Khan et al. 2011; Cai et al. 2021; Chakhtorura et al. 2023; Son and Kim 2020; Baldo 2015; Kadaj-Lipka et al. 2025; Dara et al. 2025; Salehi et al. 2019; Haddad et al. 2023; Gombart et al. 2020; Alberts et al. 2025; Lai et al. 2025; Liu et al. 2015; Fishman et al. 2000; Suvannasankha and Chirgwin 2014; Inderjeeth and Inderjeeth 2024; Marshall-Gradisnik et al. 2009; Steiner et al. 2023; Harrison et al. 2019; Chinuck et al. 2014
B – Blood and blood forming organs	Mixed classification; overlaps with A11 (vitamins), V (various), C (several), and between subgroups	missing combinations (antiplatelets, iron < 30 mg Fe^2^⁺); ambiguous assortion between B and C	“anti-” (antithrombotic, antihemorrhagic, antianemic), “other” (B06)	Galanti et al. 2024; Gupta et al. 2022; Jamali et al. 2024; Harter et al. 2015; Bereda 2022; Hoorn 2017
C – Cardiovascular system	mixed therapeutic vs mechanistic allocation; overlaps with B (several), G (urologicals), and between subgroups	missing combinations (e.g. "diuretics + "antihypertensives); missing strengths; ambiguous allocation of overlapping drugs	“blocker” (β-, α-, Ca-channel blockers), “therapy” (cardiac therapy), “mainly … effects” (C08)	Van Gelder et al. 2024; McDonagh et al. 2021; Shahn et al. 2022; Carcel et al. 2023; Kehrenberg and Bachmann 2022; Roush et al. 2014; Tang et al. 2022; Hairi and Patel 2023; Gohel and Davies 2009; Gontijo et al. 2017; Hardung et al. 2021; Diaconu et al. 2019; Sica 2006; Lee 2023; Manzar et al. 2025; Zaman et al. 2002; Pawlonka et al. 2024; Simons 2019
D – Dermatologicals	overlap with A, G, J, S, and between subgroups; overlap between systemic and local administration forms; combination preparations inconsistently classified	missing antiinfectives, GCR agonists (systemic), and antiseptics; partical coverage of topical drugs for dermatological conditions	“anti-” (antipsoriatics, antibiotics, anti-acne), “other” (D11)	Carmo et al. 2023; Hoenigl et al. 2024; Nankervis et al. 2016; Jacobi et al. 2015; Dhivya et al. 2015; Ffrench et al. 2023; Elmariah and Lerner 2011; Afifi et al. 2005; Maniyan et al. 2020; Dallo et al. 2023; Das and Panda 2017; Lata et al. 2021; Babalska et al. 2021; Williamson et al. 2017; Ghomi et al. 2019; Bhoyar et al. 2023; Mohsin et al. 2022)
G – Genito-urinary system and sex hormones	overlaps with A03 (spasmodics), L02 (hormonal cancer therapy), H (hormones)	missing antifungal/antiviral combinations; partial contraceptive coverage	“sympathomimetics” (G02), “other” (other gynecologicals)	Harper-Harrison et al. 2024; McEven and Milner 2017; Liu et al. 2011
H – Systemic hormonal preparations (excl. sex hormones and insulins)	overlaps with G (sex hormones) and A10 (insulins)	missing combinations, application forms and insulins	“therapy” (thyroid therapy H03)	Iglesias 2024; Soria et al. 2018; Raja et al. 2022; Bianco and Taylor 2024; Lee and Pearce 2023; Röder et al. 2016; Ruppe 2011; Bkaily and Jaques 2023
J – Antiinfectives for systemic use	therapeutic vs mechanistic inconsistency; overlaps with D (topical), A (intestinal), S (ophthalmic)	missing topical and combination antiinfectives	“anti-” (antibiotics, antivirals, antimycotics), “other” (J07)	Carmo et al. 2023; Hoenigl et al. 2024; Chauhan et al. 2021; Fatima et al. 2021; Fletcher et al. 2021
L – Antineoplastic and immunomodulating agents	overlaps with H (hormones), G (sex hormones), A (metabolic), J (immune sera)	missing supportive/adjuvant therapies, combination classifications, hormones, and drugs used in further conditions	“therapy” (endocrine therapy L02), “related agents” (L02A/B)	Guichard et al. 2017; Goodman et al. 2023; Olejnik et al. 2024; Patel et al. 2023; Mohi-ud-din et al. 2023; Hussain and Khan 2022; Möhlmann et al. 2024; Hosseini et al. 2019
M – Musculo-skeletal system	overlaps with N (anesthetics), R (respiratory relaxants)	missing topical and combination preparations; ambiguous listing regarding overlapping groups	“other” (M09)	Ghlichloo and Gerriets 2023; Wudexi et al. 2021; Okpala et al. 2025; Boechat et al. 2020; Vinyes et al. 2023; Beebe et al. 2005; Sattui and Gaffo 2016; McKenzie et al. 2021; Zhang et al. 2022; Tu et al. 2018; Chen et al. 2024a
N – Nervous system	overlaps with A (analgesics), R (antihistamines), M (muscle relaxants)	missing drugs used as analgesics and anaesthetics; missing administration forms; missing combination preparations	“anti-” (antiepileptics, antiparkinson, antipsychotics, antidepressants), “stimulants”, “mimetics”	Becker and Reed 2012; Vinyes et al. 2023; Kim et al. 2023; Queremel Milani and Davis 2023; Nadkarni and Devinsky 2005; Sivanandy et al. 2021; Kispotta et al. 2024; Seidel et al. 2013; Carnovale et al. 2023; Vedrenne-Gutiérrez et al. 2024; Smith Breault et al. 2025
P – Antiparasitic products, insecticides and repellents	overlaps with D (topical application), M (quinine), A (gastrointestinal tract), and between subgroups	missing infectious conditions/pathogens; ambigous listing of overlapping drugs and combination preparations	“anti-” (antiprotozoals, anthelmintics), “other”, “various”	Wozel and Blasum 2014; Sawyer et al. 1976; Sweeney et al. 2003; Martínez-Girón et al. 2008
R – Respiratory system	overlaps with A (throat/mouth), N (antihistamines), M (xanthines)	missing multi-drug cough/cold combinations; ambiguous listing for overlapping conditions	“sympathomimetics” (R01), “stimulants” (R07), “anti-” (antihistamines)	Safarov et al. 2024; van der Sandt and Ramoleta 2016; Linton et al. 2023; Cazzola et al. 2012; Restrepo 2007; Singh et al. 2025; Woo 2008; Kim et al. 2015; Schaefer et al. 2022; Hunter et al. 2022; Canonica and Blaiss 2011
S – Sensory organs	overlaps with A, D, R (local anesthetics, anti-infectives)	missing systemic application forms and combination preparations	“sympathomimetics”, “parasympathomimetics”, “indifferent preparations”	Ahmed et al. 2023; Kaushik et al. 2011
V – Various	broad overlap with most groups; residual for unassigned drugs	Missing dietetic, diagnostic, and topical drugs	“general”, “other”, “various”	

### B- blood and blood forming organs

This group focuses on drugs related to blood and haematopoiesis, with categories based on pharmacologic, therapeutic, and miscellaneous aspects. The presence of the catch-all “other” (B06) indicates structural insufficiency. An overview of exemplary omitted drug classes in the respective main categories can be found in Table 3.

**Table 3:** Examples illustrating omitted drug classes within the ATC main groups that are not represented as separate categories. Notably, the listed drug classes are often not limited to therapeutic use within their respective main group

Main group	Omitted drug classes (examples)	Source
A – Alimentary tract and metabolism	NTCP inhibitors, FXR agonists, microbiome-based therapeutics	Tan et al. 2024; Almeqdadi and Gordon 2024; Pitashny et al. 2025
B – Blood and blood forming organs	HIF-prolyl hydroxylase inhibitors, hepcidin antagonists, PAR-1 antagonists	Haase 2021; Poli et al. 2014; Sagar et al. 2021
C – Cardiovascular system	PCSK9 inhibitors, soluble guanylate cyclase stimulators, angiopoietin-like protein inhibitors	Vahdat-Lasemi et al. 2025; Gawrys et al. 2025; Nair 2024
D – Dermatologicals	topical JAK inhibitors, topical TYK2 inhibitors, topical HDAC inhibitors	Dogra et al. 2023; Martin 2023; Jin et al. 2024
G – Genito-urinary system and sex hormones	beta 3 agonists, selective progesterone receptor modulators	Schena and Caplan 2019; Critchley and Chodankar 2020
H – Systemic hormonal preparations (excl. sex hormones and insulins)	dual/triple incretin receptor agonists, long-acting growth hormone analogues, glucocorticoid receptor blockers	Alfaris et al. 2024; Zhu et al. 2024; Molitch 2022
J – Antiinfectives for systemic use	bacteriophage therapy, host-directed antivirals, RNA-based antivirals	WHO 2025a; Nizi et al. 2025; Shi et al. 2024
L – Antineoplastic and immunomodulating agents	androgen receptor degraders (AR PROTACs), CAR-T therapies	Chen et al. 2024b; Zugasti et al. 2025
M – Musculo-skeletal system	antisense oligonucleotides for muscular dystrophies, sclerostin inhibitors	Egli and Manoharan 2023; Yu et al. 2022
N – Nervous system	anti-amyloid monoclonal antibodies, RNA therapeutics, neuro-degraders (PROTACs)	Gregory et al. 2024; Kim et al. 2025; Anthony 2022
P – Antiparasitic products, insecticides and repellents	host-directed antiparasitic small molecules, cyanotriazole compounds	Aphasizhev and Aphasizheva 2023; Jans and Wagstaff 2021
R – Respiratory system	gene therapies, small-molecules	Kingwell 2025; Flotte 2025
S – Sensory organs	VEGF inhibitors, ocular gene therapies	Krajewska and Waszczykowska 2025; Amadio et al. 2016
V – Various	-	

Overlaps are particularly strong with “cardiovascular system” (C) and “various” (V), as well as internally across B01–B06 (Table 2 and S1). Combination handling is inconsistent, as seen in the exclusion of “local haemostatics” (A01) from “antihemorrhagics” (B02) versus the inclusion of acetylsalicylic acid (N02) plus rivaroxaban in B01AF. Exceptions to ATC principles also occur, e.g. salicylic acid receiving more than one ATC code per route of administration (WHO 2025b).

### C- cardiovascular system

This group targets cardiovascular conditions. The classification shows strong internal overlaps, leading to incomplete therapeutic areas (Table 2 and S1). For example, “antihypertensives” (C02) do not include all relevant drug classes, which are spread across “peripheral vasodilators” (C04), “vasoprotectives “ (C07), “calcium channel blockers” (C08), and “agents acting on the renin-angiotensin system” (C09).

Externally, overlaps with “alimentary tract and metabolism” (A), “blood and blood forming organs” (B), and others, are frequent, e.g. prostaglandins (listed in A02, C01, G02, and S01). Cross-classification issues also arise, such as antiarrhythmics (C01BA, C01BB, C01BG). Combination handling is inconsistent, and exclusions contribute to fragmented coverage.

### D- dermatologicals

This group addresses dermatological conditions, including many systemic-use drugs despite the topical focus. It comprises subgroups with varying classification bases, and the presence of a miscellaneous group (D11) underlines structural gaps.

Overlaps with other main groups are pronounced due to the broad therapeutic range, visible already in redundant titles like “antibiotics and chemotherapeutics” (D06) or “corticosteroids” (D07) (Table 2 and S1). Not all dermatological drugs are included, and application route (topical/local/systemic) is inconsistently regarded. Internal overlaps exist, e.g. between “antifungals” (D01), “wound/ulcer treatments” (D03), “antibiotics” (D06), “antiseptics” (D08), and others. Cross-classification failures are illustrated by “corticosteroid” combinations and exceptions, where the combinations are listed not in the “corticosteroid” chapter and are even excluded from the main group, e.g. “antifungals” (D01A) or “antihemmorhoidals” (C05A).

### G – genito urinary system and sex hormones

This group covers gynaecologic, urologic, and sex hormone-related treatments. Substantial overlaps exist with “systemic antiinfectives” (J), “antineoplastic and immunomodulating agents” (L), and “systemic hormonal preparations” (H) (Table 2 and S1).

This results in incomplete subgroups, e.g. by the exclusion of alpha-adrenoreceptor blockers (C02CA) from “urologicals” (G04), or antivirals for topical use (D06) from “gynecological antiinfectives and antiseptics” (G01). Internal overlaps between G01–G04 cause unrelated inclusions or omissions, e.g. for contraceptices for systemic use being classified in “sex hormones” (G03A), which could also belong in “other gynecologicals” (G02). Combination classification is inconsistent, as in seen in trospium plus analgesics appearing in A03DA.

### H- systemic hormonal preparations, excl. sex hormones and insulins

This group includes hormonal preparations for systemic use, but with many exclusions, including “insulins” and “anabolic steroids” (A), “catecholamines” (C, R) “sex hormones” (G, L), and others. Consequently, coverage is incomplete and overlaps are common (Table 2 and S1).

“Corticosteroids” (H02) illustrate inconsistency. Although all systemic corticosteroids should be listed here, combinations with “anti-inflammatory/antirheumatic drugs” are excluded (classified in M01BA). Additional gaps appear for drugs used diagnostically, e.g. “pituitary and hypothalamic hormones” in V04. Internal overlaps occur with G subgroups, such as intrauterine hormone devices listed under “other gynecologicals” (G02B) instead of “sex hormones”.

### J – antiinfectives for systemic use

This group covers bacterial (J01), viral (J05), fungal (J02), and mycobacterial (J04) infections, plus vaccines (J07) and immunoglobulins (J06), but excludes parasites (P). Connections to other main groups are extensive, since infections occur across all organ systems (particularly redundant in A, D, G, P, R, S).

The focus is on systemic drugs, with a large number excluded for topical or local use (Table 2 and S1). However, systemic use can also be excluded, for example in the case of “antimycotics for dermatological use” (listed in D01B instead of J02). Additionally, the use of systemic drugs can overlap, for example due to various drug indications. This results in an incomplete listing when drugs are listed elsewhere, e.g. thalidomide is listed under L04AX instead of J04. Furthermore, there are overlaps between subgroups. For example, “antibacterials” (J01) and “antimycobacterials” (J04) intersect, and although streptomycin is indicated for both pathogens, it is only listed under J01G. Additionally, combination preparations are handled inconsistently across J01-J07 and with other main groups. For example, antibacterials plus local anaesthetics are classified under J01R, despite including additional, not labeled drugs in plain drug groups. For instance, isoniazid plus rifampicin plus other tuberculostatics are classified under J04AM, while some combinations are classified in separate combination groups.

### L- antineoplastic and immunomodulating agents

This group targets neoplastic and immunological conditions, including antineoplastics (L01), endocrine therapy (L02), immunostimulants (L03), and immunosuppressants (L04). This results in the overlap with other main groups, particularly with hormones (G, H), resulting in the exclusion of corticosteroids (listed in H2).

Therapeutic areas and drugs overlap across the subclassifications L01-L04 (level 2), as well as in their ATC levels 3 and 4 (Table 2 and S1). For example, “antineoplastic agents” (L01) overlap with “immunosuppressants” (L04), which includes sirolimus and CD20 inhibitors. This results in excluded drugs and the confusing handling of combination preparations. Groups are often incomplete: for example, “endocrine therapy” (L02) excludes “antigrowth hormones” (listed in H01BC), while the combination of polyestradiol with "non-listed" local anaesthetics is included (L02AA). As there are no separate categories for combination preparations, they are included in the plain drug categories. For example, both JAK inhibitors and TYK2 inhibitors are included in "JAK-inhibitors" (L04AF).

### M – musculo-skeletal system

This group addresses muscle and bone conditions across diverse therapeutic areas. A miscellaneous category (M09) reflects structural gaps. Overlaps occur with subclassifications and many other main groups, particularly hormones (G, H), minerals (A), and topical preparations (D, G).

Cross-indication is common (Table 2 and S1). For example, “anti-inflammatory and antirheumatic products” (M01) overlap with “analgesics” (N02, e.g. ibuprofen), “hormonal preparations” (H, e.g. corticosteroids), “topical products” (M02, e.g. capsaicin), and miscellaneous entries (M09). This leads to incomplete coverage, as some relevant preparations are classified elsewhere, e.g. sugammadex listed as an antidote in “various” (V03AB).

Combination handling is also inconsistent. Sometimes, there are separate combination categories (M01BX, M05BB). However, other combinations are placed in plain substance groups, such as “ethers” (M03BC) combined with paracetamol in M03BC.

### N – nervous system

This group covers drugs for nervous system conditions or those exerting neurological effects. Its broad therapeutic scope creates many overlaps with other main groups and internally across N01–N07 (Table 2 and S1). The presence of a miscellaneous “other” (N07) signals classification limitation.

Many subgroups are incomplete, such as “anesthetics” (N01) and “analgesics” (N02), due to allocation in other therapeutic areas (e.g. N02 fentanyl in sublingual form, benzodiazepines in N05). Combination handling is not systematically addressed, contributing to inconsistency. For example, the combination fentanyl plus bupivacaine in plain “opioid anesthetics” (N01AH), versus anesthetics plus stomatologicals in “stomatological preparations” (A01).

### P – antiparasitic products, insecticides and repellents

This group includes “antiprotozoals” (P01), “anthelmintics” (P02), and “ectoparasiticides, scabicides, insecticides and repellents” (P03). Subdivision by species leads to incomplete groups, with only selected pathogens covered.

Overlap with other main groups is common, since antiparasitic agents have multiple uses and are often used in combination (Table 2 and S1). They are often excluded and listed in other groups, for example, clioquinol is used for dermatological purposes and is listed under dermatologicals (D08AH), while dimeticon is used as an antiflatulent and is listed under “functional gastrointestinal disorders” (A03AX). Combination preparations are handled inconsistently and do not have separate categories. While some combinations of drugs are excluded and listed elsewhere, e.g. antiparasitics plus antibacterials in “antibacterials” (J01R), other “plain” drug groups include combinations with other drugs, e.g. benzyl benzoate plus sulfur in “ectoparasiticides” (P03AA).

### R- respiratory system

This group addresses respiratory conditions, structured partly by application site (R01 nasal, R02 throat) and partly by condition (R03 obstructive airways, R05 cough/cold) or pharmacology (R06 histamine receptor antagonists). A miscellaneous category (R07) is also present. “Inhaled antiinfectives” are excluded and placed in J.

There are overlaps for all subgroups, including redundancies across R01-R07 and with other main groups (Table 2 and S1). For example, there is overlap between “antihistamines for systemic use” (R06) and “nasal preparations” (R01) for histamine receptor antagonists. Categorisation by application area results in incomplete and redundant groups for drugs that may be allocated in various ways, particularly within the main group “sensory organs” (S), e.g. varenicline nasal spray (S01XA).

Combinations are handled inconsistently. While a few combinations have separate categories, they are often listed in 'plain' drug groups, e.g. corticosteroids plus anti-infectives (R01AD), and mucolytics plus anti-inflammatories (M01).

### S – sensory organs

This group focuses on “ophthalmologicals” (S01), “otologicals” (S02), and preparations for both eye and ear (S03). It does not cover all sensory organs, excluding skin, nose, and mouth. Overlaps occur between S01–S03 and with other main groups, leading to missing entries in S01 and S02 when drugs are classified in S03 (Table 2 and S1).

Some drugs are excluded entirely, e.g. mitomycin (L01) for ophthalmological use or corticosteroids for otological use. Although a number of combination groups exist, others are integrated into plain categories, e.g. antibacterials plus other drugs in “ophthalmologicals” (S01AA).

### V—various

This heterogeneous group contains drugs that cannot be allocated to a defined therapeutic or anatomical category. Subgroups are mostly unrelated. However, a few groups overlap, e.g. “diagnostic agents” (V04), “contrast media” (V08), as well as “diagnostic” (V09) and “therapeutic” (V10) “radiopharmaceuticals”. Another miscellaneous subgroup exists (V07).

Due to its catch-all nature, overlaps with other main groups are numerous, and subcategories are often incomplete, e.g. exclusion of “parenteral nutrition” (B05BA) from “general nutrients” (V06) (Table 2 and S1).

## Discussion

### Inconsistencies in the ATC system

Inconsistencies refer to internal conflicts within the ATC classification, including its structural logic, classification principles, and methodology. Identified issues include the mixing of classification criteria within and across ATC levels, unclear application of definitions such as “therapeutic” or “pharmacological,” overlaps between therapeutic areas, use of catch-all categories like “other/miscellaneous”, and inconsistent handling of combination products.

A major inconsistency lies in the mixing of classification principles (anatomical, therapeutic, pharmacological, chemical) (Nelson et al. 2017) across an ATC level (Table 4). For instance, Level 1 Group M refers to an anatomical system (“musculo-skeletal”), while Group L is therapeutic (“antineoplastic and immunomodulating agents”). Within Group M, Level 2 subgroups are based on indication (M04 “antigout”), pharmacological action (M03 “muscle relaxants”), or remain undefined (M09 “other”). This lack of structural consistency complicates interpretation (Günther et al. 2025).

**Table 4:** Overview of major inconsistencies, insufficiencies, and terminology issues identified across ATC Levels 1–4

Criteria	ATC Level 1	ATC Level 2	ATC Level 3	ATC Level 4
Inconsistencies (Nelson et al. 2017; WHO 2024, 2018; Winnenburg and Bodenreider 2013, 2014; Serafini et al. 2021)	Mixed principles across broad anatomical groups (e.g. anatomic vs. therapeutic); overlap with other anatomical groups	Mixed classification logic within groups, e.g., combining therapeutic and chemical classifications at the same ATC level 2 group; inconsistent handling of combination drugs across subgroups	Shifts in logic within subgroups: Level 3 sometimes organized by mechanism, sometimes by therapeutic indication, causing confusion	Combination products inconsistently classified (sometimes in new subgroup, sometimes merged, sometimes missing); exceptions in allocation (see WHO guideline)
Insufficiencies (WHO 2024, 2018; Ludwig et al. 2025; Chen et al. 2018)	Some relevant drug classes or therapies missing from entire anatomical groups; lack of representation for new or emerging drug classes; presence of category "V" without logic	Incomplete subgroup coverage, missing key therapeutic classes or chemical subgroups; insufficient granularity for certain drug types; category "other"	Lack of uniform subgrouping structure; some groups lump diverse drugs under “other” with little specification	Failure to account for all relevant combinations, routes, or formulations; missing drug forms; inadequate cross-indication classification
Terminology Issues (Seifert and Alexander 2022; Winnenburg and Bodenreider 2014; Robertson et al. 2019)	Level 1 group names sometimes overly broad or ambiguous (e.g., “alimentary tract and metabolism”); inconsistent use of anatomical terms vs. therapeutic descriptions	Group names at Level 2 occasionally vague or outdated (e.g., “Other systemic drugs”, "antiepileptics"); mixture of pharmacological and therapeutic terms without clarification	Subgroup titles sometimes unclear or overlapping in meaning; inconsistent use of pharmacological vs therapeutic terminology	Subgroup names may be outdated, too broad, or use terms inconsistent with modern pharmacology or clinical practice; “other” categories prevalent

The presence of "other/miscellaneous" categories at multiple levels (WHO 2018, 2024) highlights the system’s limitations to classify certain drugs within its existing framework (Table 5). These residual groupings reflect insufficiencies in classification logic rather than functional categorisation by mixing unrelated substances (Sathyanarayana Rao and Andrade 2016).

**Table 5:** Examples of drugs and drug classes that are listed in the ATC system under a specific therapeutic area, despite being used for a wide range of conditions and in many different fields. As it is not possible to distinguish between indications in consumption data (Ludwig et al. 2025), the classification by usage cannot be reasoned

Drug class	Drug (example)	Therapeutic listing in the ATC system	Indications/usage fields	Source
Benzodiazepines	diazepam	"antiepileptics" N03A	anaesthesia, anxiety disorders, skeletal muscle spasms, adjunct in epilepsy therapy	Olkkola and Ahonen 2008; Abd-Elsayed et al. 2024; Calcaterra and Barrow 2014
Gabapentinoids	gabapentin	"other analgesics" N02B	epilepsy, postherpetic neuralgia, peripheral/diabetic neuropathic pain, restless legs syndrome, anxiety disorders (wide off-label use)	Gupta 2023; Panebianco et al. 2021; Greenblatt and Greenblatt 2018; Irving 2012; Mayo-Wilson et al. 2021; Heidbreder et al. 2023; Bindel and Seifert 2025c
SGLT2 inhibitors	empagliflozine	"drugs used in diabetes" A10B	heart failure, chronic kidney disease, diabetes type 2	Padda et al. 2023; Talha et al. 2023; Yau et al. 2022; Ludwig et al. 2025; UKKA 2022
Vinca alkaloids	colchicine	"antigout preparations" M04	gout, cardiovascular event prevention	Buckley and Libby 2024
Monoclonal antibodies	dupilumab	" other dermatological preparations" D11A	respiratory diseases (asthma, COPD); dermatology (atopic dermatitis, prurigo nodularis), gastrointestinal tract (eosinophilic esophagitis), sensory organs (chronic rhinosinusitis)	Ludwig et al. 2025; IQWiG 2018
JAK inhibitors	baricitinib	"immunosuppressants" L04A	dermatology (psoriasis, pruritis, lupus erythematosus, dermatomyositis, …), musculo-skeletal system (rheumatoid arthritis, …), gastrointestinal tract (inflammatory bowel disease, …), heamatology/oncology (myeloproliferative neoplasms, …)	Chikhounde et al. 2025; Shawky et al. 2022
mGPCR antagonists	olanzapine	"antipsychotics" N05A	schizophrenia, moderate—severe manic episodes, bipolar disorder, anxiety	Biso et al. 2025; Christian et al. 2012
Dopamine agonist	levodopa	"anti-parkinson drugs" N04B	parkinson, restless legs syndrome	Heidbreder et al. 2023; EMA 2025; Trenkwalder et al. 2024

Overlap between therapeutic areas leads to ambiguity, omissions, redundancies and inconsistent listing (Winnenburg and Bodenreider 2013, 2014; Nelson et al. 2017; Chen et al. 2018; Liang et al. 2020). Some drugs span multiple indications but are assigned to only one ATC group or code (Günther et al. 2025), while others appear in several (WHO 2024, 2025b). This results in fragmented or duplicated representation, as seen with “corticosteroids” and “antibacterials” (Table 2 and S1). Combination products are also handled inconsistently, with the inclusion in plain substance groups, exclusion of the therapeutic group, or the introduction of separate combination categories, e.g. in HIV drugs (WHO 2024). The presence of modified ATC systems for national use, as seen in Germany (Günther et al. 2025) (Table S2 and S3), illustrates the ambiguity and the indiscriminate categorisation of groups and allocation of drugs.

Overall, the system’s classification logic is opaque (Winnenburg and Bodenreider 2014; Sathyanarayana Rao and Andrade 2016). Terms like “anatomical,” “therapeutic,” or “pharmacological” are not clearly defined and are applied inconsistently across and within groups (Seifert 2018a, 2018b; Winnenburg and Bodenreider 2014). Understanding classification decisions often requires the explanation of the ATC guideline. Special cases, such as the classification of acetylsalicylic acid (WHO 2025b), illustrate arbitrary exceptions that undermine internal coherence and the methodology principles (Table 1).

### Insufficiencies in the ATC system

**Fig. 3:**
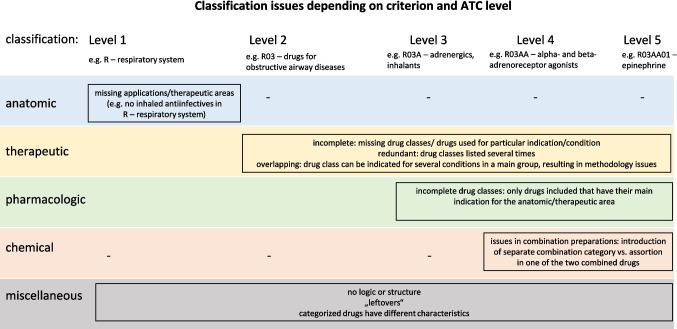
Overview of classification issues depending on criterion (anatomic, therapeutic, pharmacologic, chemic, miscellaneous), and ATC level (1–5)

Insufficiencies concern areas where the ATC classification is incomplete, narrow, or fails to reflect current pharmacological practice. Main issues include missing drug classes or individual drugs, exclusion of relevant indications or routes of application, the handling of drugs with multiple indications, and incomplete listing of available drugs (Fig. 3). These insufficiencies reduce the system’s accuracy and completeness.

Drugs with multiple indications are frequently classified under only one therapeutic area (Günther et al. 2025), hiding their broader use. For example, benzodiazepines are listed solely under “antiepileptics” (N03AE), despite their established role in anaesthesia (Olkkola and Ahonen 2008; Abd-Elsayed et al. 2024). Gabapentinoids appear only under “analgesics” (N02BF), although they are also used in epilepsy (Gupta 2023; Panebianco et al. 2021), SGLT2 inhibitors are listed as “antidiabetics” (A10BK), despite their approved use in heart failure and chronic kidney disease (Padda et al. 2023; Talha et al. 2023; Yau et al. 2022; Ludwig et al. 2025). This list extends to many other drugs (Table 5). Pharmaceutical companies might misuse this procedure by placing their drug in a group that is beneficial for approval, reimbursement, or marketing. In general, it does not make sense to categorise drugs by therapeutic field, as it is not possible to differentiate consumption volumes for different indications (Ludwig et al. 2025). Furthermore, this situation can lead to misinterpretation, since drugs assigned to one usage field in the ATC system are often used in more than one indication. This problem can be partly solved by differentiating consumption volumes by prescriber group (Rasmussen et al. 2022), which helps distinguish between usage fields. However, this method is only applicable to drugs for which different medical specialities treat different indications.

Fragmentation of drug classes is another key issue. Antibacterials, though grouped in ATC J, also appear in various other groups depending on application or therapeutic use (e.g. A, C, D, G, L, R, S). This dispersal divides pharmacologically related drugs, masking their comprehensive use. Similar duplication appears in overlapping drug groups (Chen et al. 2018), such as ramipril (C09 and C10) or heparin (B01, C05, S01), leading to inconsistencies in corresponding classifications. While some drugs have multiple ATC codes assigned to different application forms (e.g. salbutamol, R03AC02 vs. R03CC02), others have only one ATC code assigned to several application forms (e.g. ibuprofen, M01AE01) (WHO 2024). In general, there is no overarching category where consistency or completeness is achieved, neither within a main group, a therapeutic group, or a pharmacological subgroup.

Moreover, substances with distinct mechanisms may be grouped together despite lacking shared pharmacology (Serafini et al. 2021; Seifert and Alexander 2022). For example, esketamine (NMDA antagonist) and mirtazapine (NaSSA) are both classified under “other antidepressants” (N06AX), despite significant pharmacological differences (Kawczak et al. 2024; Vasiliu 2023; Anttila and Leinonen 2001). Conversely, drugs with related characteristics are split across categories, e.g. gabapentinoids and “other antiepileptics” (N02BF vs. N3AX), reducing therapeutic clarity.

### Terminology issues in the ATC system

Issues in the chosen names for ATC groups refer to terminological problems that cause confusion, misinterpretation, or misrepresentation. These include vague, broad, outdated, or imprecise terminology, inconsistent naming conventions, and classification labels that do not reflect their content (Robertson et al. 2019; Serafini et al. 2021; Seifert 2018a; Winnenburg and Bodenreider 2014) (Table 6).

**Table 6:** Issues in ATC group titles and nomenclature. Presented are the problematic principles/terms, along with examples of ATC groups and an explanation of the problematic aspect, followed by a suggestion of a more appropriate replacement

Problematic term	Example from ATC classification (WHO 2024)	Explanation of issue	Rational replacement (Neubig et al. 2003; Seifert 2018a, 2018b; Serafini et al. 2021; Robertson et al. 2019; Seifert and Alexander 2022)
anti- (regarding therapeutic use, not pharmacological effect)	antidiarrheals (A07) antiobesity (A08), antithrombotic (B02), antihemmorhagics (B02), antianemic (B03), antipsoriatics (D05), antibiotics (D06), anti-acne (D10), antiepileptics (N03), anti-parkinson (N04), antipsychotics (N05), antidepressants (N06), …	imprecise, vague, allocation of drugs with various mechanism of actions, therapeutic areas and characteristics, defined by intended effect, not mechanism implies a single direct action	mechanism-based nomenclature (e.g. mGPCR antagonist instead "antipsychotics/neuroleptics"); neutral description (e.g. antagonists or inhibitors instead "anti")
blocker	calcium channel blockers (C08), beta blockers (C07), alpha blockers (C07), angiotensin receptor blockers (C09)	imprecise, colloquial, no interference on mechanism of action	inhibitors, antagonists (e.g. beta-adrenergic receptor (beta-x-AR) antagonists for "beta blockers")
stimulants	appetite stimulants (A15), immunostimulants (L03), cardiac stimulants (C01), psychostimulants (N06), respiratory stimulants (R07)	imprecise, colloquial, no interference on mechanism of action	agonists/mechanism-based nomenclature
mimetics	sympathomimetics (G02, N06, R01, S01), parasympathomimetics (N07, S01)	imprecise, too general	description of mechanism of action (e.g. muscarinic receptor (MxR) agonists for directly acting "parasympathomimetics"; acetylcholine esterase inhibitors (AChEI) for indirectly acting "parasympathomimetics"; alpha-2 adrenoceptor (alpha2AR) antagonists for "sympathomimetics")
therapy	bile and liver therapy (A05), cardiac therapy (C01), thyroid therapy (H02), endocrine therapy (L02)	imprecise, no information about listed drugs, their characteristics, pharmacological effects or mechanism of action; listed drugs often have several indications	neutral description, subclassified by pharmacologic mechanism of action (e.g. "drugs influencing the cardiovascular system" instead "cardiac therapy")
general, indifferent	general nutrients (V06), anesthetics general (N01), other general anesthetics (N01), indifferent preparations (S02)	imprecise, too broad, unclear meaning	precise and neutral title (e.g. "anesthetics having an effect on the complete organism" instead "anesthetics, general")
mainly … effects	selective calcium channel blockers with mainly vascular effects (C08)	imprecise, incomplete, misleading	classification on mechanism of action (e.g. L-type calcium channel inhibitors)
other, various	other alimentary tract and metabolism products (A16), other hematological agents (B06), other dermatological preparations (D11), other gynecologicals (G02) other drugs for disorders of the musculo-skeletal system (M09), other nervous system drugs (N07), other respiratory system products (R07), all other therapeutic products (V03), all other non-therapeutic products (V07), …, various alimentary tract and metabolism products (A16AX), various (V, A01AB, A01AD, M02AX, R01AX, R02AA), …	undefined, no structure, no interference to any characteristics	classification by mechanism of action, pharmacological effect or chemical properties
related agents	hormones and related agents (L02A), hormone antagonists and related agents (L02B)	imprecise	classification on mechanism of action of chemical structure, introduction of combination classifications
cardiac glycosides	C01A	description of chemical components is linked to pharmacokinetics but not the primary effect; variability of pharmacological characteristics along (cardiac) glycosides	Na +/K + ATPase inhibitors (name defined by mechanism of action)
diuretics (thiazide, loop)	C03	imprecise, description of functional effect while different mechanisms of action, therapeutic range exceeds diuresis	inhbitors of Na +/Cl- (thiazide)/Na +/K +/2Cl- (thiazide) cotransporter (NCC; NKCC inhibitors)
non-opioids	N02A	vague, too broad, negative stigma	non-MOR agonists, subdivided by precise naming of mechanism of action
opioids	N02A	vague, too broad, negative stigma	µ-opioid receptor (MOR) agonists

A major problem in the ATC classification system is the use of vague, overly broad, misleading, or outdated group titles that hinder clear understanding and scientific accuracy (Günther et al. 2025). Terms such as “other analgesics” offer little insight into pharmacological characteristics or classification structure. Furthermore, group labels such as “antiepileptics,” “antibiotics,” or “beta-blockers” reflect historical names rather than scientific accuracy (Table 6), often failing to account for multi-indicational use, precise indications, or mechanisms of action (Seifert 2018a; Taddei et al. 2024). Similarly, many classifications, despite appearing structured, remain opaque, e.g. “cardiac therapy”, with their logic unclear or counterintuitive (Nelson et al. 2017). In many cases, the rationale for why substances are grouped together cannot be derived from the group name itself but requires consulting WHO guidelines, reinforced by a poor web page presentation with an incomplete listing of included drugs. This further limit the self-explanatory understanding of the system.

Naming conventions are applied inconsistently. Some groups are defined by indication, others by pharmacological mechanism, chemical structure, or therapeutic effect (Winnenburg and Bodenreider 2014). Furthermore, some drugs are referred to with different names, e.g. “antibacterials” (J01, J06) vs. “antibiotic” (D06, A07, C05, G01, …). This inconsistency reduces comparability between groups and weakens the system’s internal logic. Moreover, the ATC system continues to rely on outdated terminology (Seifert 2018a; Serafini et al. 2021; Robertson et al. 2019). Many terms do not reflect contemporary therapeutic concepts such as drug repurposing, polypharmacy, or combination therapies.

The widespread use of “anti-” prefixes (e.g. “antidepressants,” “antidiabetics,” “antibiotics”) reinforces a narrow and negatively framed understanding of drug function, ignoring the broader or multiple uses of many agents. The problem is compounded by the practice of naming drug groups by its initial indication, such as “antidepressants” or “diuretics”, even though these drugs are frequently used across several clinical contexts or have changed their primary indication (Alvano and Zieher 2020; Kehrenberg and Bachmann). This creates misalignment between classification and real-world use, limiting interpretability and relevance. Table 7 summarizes key literature of the discussed aspects of the ATC system.

**Table 7:** Selection of key literature with regard to issues of the ATC system

Reference	Aspect	Key findings
Filippo Caraci et al. 2017	nomenclature	neuroscience-based nomenclature (NbN) developed as an alternative to ATC; limited nomenclature for psychotropics by ATTC because pharmacological effects/mechanism of action, does not describe all potential uses of a particular drug; disconnection between drug classification and clinical use; problematic in scientific purposes and causing confusion for patients and professions; leads to misunderstanding of intended effects; consequence is low treatment adherence; improved and more rational classification is based on mechanism of action
Günther et al. 2025	structure and consistency; differences in the German ATC system	additions and extensions of the international ATC system for Germany, because of relevant gaps for the German pharmaceutical market; broad classification of therapeutic areas; many drug preparations used for two or more similarly important indications, but assignation of only one ATC code; one ATC group may summarize drugs with various indications vs. drugs with similar indications may be listed in different groups; main indication of a drug may vary within countries, resulting in multiple options for allocation; grouping of combination preparations is inconsistent and a problem, with outdated classifications; exceptions of the ATC methodology are present
Nelson et al. 2017	structure	each classification with different grouping criteria; “comparing and mapping between medication classes is challenging task due to the different ways of classifying medications”
Sathyanarayana Rao and Andrade 2016	structure	allocation of drugs to anatomic system not possible for all drugs since universal for systems by acting at a molecular target (e.g. antibacterials, antineoplastic drugs, vitamins); problem of assigning drugs to class of their original indication for which they were approved, while having many indications (e.g. SSRIs efficient in depression, anxiety disorder, migraine, …); unsystematic subclassification with different classification criteria (mechanism, structure, miscellaneous); indication-based structure introduces stigma; application of a scientifically classification (updateable, no conflict with therapeutic indications, assisting clinicians in clinical practice)
Seifert 2018a	nomenclature	many common terms are outdated; need of mechanism-based nomenclature to support rational prescribing and avoid confusion; several problematic terms are used and have to be replaced by a precise and rational drug name (e.g. NSAID by COX inhibitor); long-lasting process to change established "irrational" nomenclature
Seifert and Alexander 2022	nomenclature	classification of drugs by therapeutic areas due to obscure mechanism of action in the past; change in use of drugs causes confusion; outdated names can lead to misconceptions (e.g. "antibiotic" suggesting that effective against any pathogens while only being effective against bacteria); improvement of therapeutic and chemic drug names towards mechanistically orientated nomenclature; advantages of correct and consistent use, simplified teaching to healthcare professionals, improvements in drug safety and knowledge in patients; challenges to adopt a new nomenclature while the outdated one is commonly used
Serafini et al. 2021	nomenclature	INN name for drug description usually vague and broad, “it may refer to its chemistry (e.g., vitamin D analogue), to its pharmacological action (e.g., antiviral), or to its mechanism of action (e.g., toll-like receptor antagonist)”; “drugs undergo an extension of indications that would not be represented in the initial description”
Winnenburg and Bodenreider 2013	overlaps	homogenity is considered when drugs have similar characteristics; ATC methodology states that “Substances classified in the same ATC fourth level cannot be considered pharmaco-therapeutically equivalent since their mode of action, therapeutic effect, drug interactions and adverse drug reaction profile may differ"; less homogenous in therapeutic categories (e.g. L01, B05, D03) vs. well homogenous in mechanism of action (e.g. J01, D07, A10)
Winnenburg and Bodenreider 2014	consistency	ATC has a complex classification principle with mixed criteria (anatomic, therapeutic, pharmacologic, chemic); no unique name for a class, can refer to different classes, e.g. fluoroquinolones (S, J); discrepancies in MeSH descriptors and missing assignations; underspecified name of ATC class, “derives part of its meaning from its position in the hierarchy”;

## Conclusion: overarching problems in the ATC classification

**Fig. 4:**
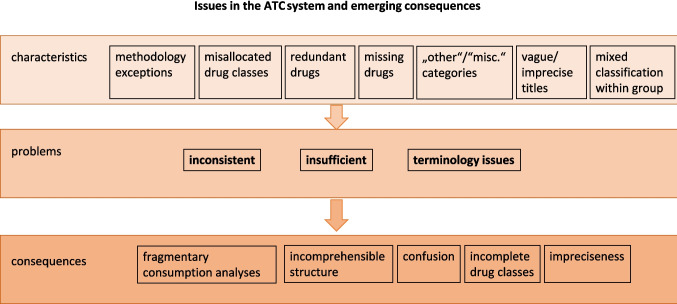
Problematic characteristics of the ATC system, their relating aspects, and resulting consequences

Despite its central role in global drug monitoring, the ATC classification system presents fundamental structural and conceptual shortcomings that limit its adequacy pharmacological classification and consumption analysis (Fig. 4). Its hierarchical structure was designed to provide clarity through an anatomical-to-chemical cascade. While this has historically been appropriate and useful, issues with classification have emerged, resulting in a fragmented and inconsistently applied system whose logic often fails under scrutiny (Winnenburg and Bodenreider 2013. The resulting problems, confusion, imprecision, incompleteness, are not incidental exceptions but are present systematically throughout the whole ATC system (Tables 2, 4 and S1).

A key problem lies in the underlying principle of classification itself. The ATC system prioritizes single indications, resulting in heterogenous drug groups (Winnenburg and Bodenreider 2014; Nelson et al. 2017). The ATC system was developed at a time when drugs were typically assigned to single indications and grouped into relatively simple categories, which provided a sufficient framework for classification some decades before (Günther et al. 2025). In contrast, modern pharmacotherapy is increasingly characterised by polyfunctionality (Ryszkiewicz et al. 2023), with drugs having multiple effects, several indications (Foucquier and Guedj 2015), and differing therapeutic use across countries (Günther et al. 2025). Furthermore, many more pharmacological classes have been developed over the last years that need acknowledgement. The ATC framework has not adapted to this complexity. By continuing to force drugs into therapeutic, single-indication, categories, it fails to reflect actual clinical use (Winnenburg and Bodenreider 2014; Günther et al. 2025). Due to this undifferentiated classification (Chen et al. 2024a, 2024b), the analysis of consumption volumes is restricted (Ogorek et al. 2025). It creates direct complications in data interpretation, such as over- or underestimating consumption, misrepresenting therapeutic priorities, and concealing shifts in prescribing (Ludwig et al. 2025).

Closely tied to this is the system’s limited capacity to adapt on innovation in medicine and pharmacotherapy. While new drugs continue to enter the market and older drugs are repurposed and used for new indications, the ATC structure remains unchanged (WHO 2018). Furthermore, globally withdrawn drugs are maintained (Table 8), while approved drugs lack an ATC code until a request is sent because drugs are not automatically assigned to an ATC code (WHO 2025d; Wang et al. 2013; Wikipedia 2022), resulting in the omitting of these prescriptions in drug consumption statistics (Mühlbauer et al. 2009). This stagnation is most evident in the continued introduction of vague or catch-all categories, which summarized innovational, but pharmacologically unrelated drugs under “miscellaneous/other” (WHO 2024, 2025b). The consequences are significant: analytical granularity is lost, cross-national comparisons become less meaningful, and classification-based drug surveillance, even for defined drug classes, becomes unreliable. In many therapeutic areas, miscellaneous categories and listed drugs became extensive (e.g. L01X “other antineoplastic agents”) (Table 9).

**Table 8:** Examples of globally withdrawn drugs (Onakpoya et al. 2016; Withdrawn 2.0, 2023) still listed in the ATC system (WHO 2025a)

Withdrawn drug	ATC code	Main group ATC system
benoxaprofen	M01AE06	M—Musculo-skeletal system
cerivastatin	C10AA06	C—cardiovascular system
chlormezanone	M03BB02	M—Musculo-skeletal system
fenfluramine	A08AA02	A- Alimentary tract and metabolism
glafenine	N02BG03	N—Nervous system
indoprofen	M01AE10	M—Musculo-skeletal system
temafloxacin	J01MA05	J—Antiinfectives for systemic use

**Table 9:** Summary of overarching problems in the ATC system and corresponding solutions proposed through a mechanistically-oriented nomenclature

Category	Issue	Manifestation	Proposal for Improvement (mechanism-oriented classification & nomenclature)
Classification logic	Inconsistent classification logic	Mixed principles (therapeutic area, chemical identity, pharmacologic effect, mechanism of action, undefined) within/across ATC levels (Nelson et al. 2017; Winnenburg and Bodenreider 2013)	Primary classification strictly by dominant mechanism of action
	Misallocated drugs	Outdated assignments, unclear rules for combinations, exceptions for single drugs, other drug classification possible (WHO 2024, 2018; Ludwig et al. 2025; Liang et al. 2020)	One defined group per drug; objective allocation criteria
	Redundant/overlapping classification	Same drug in multiple ATC groups without rationale vs. missing drugs in indicated therapeutic areas (Chen et al. 2018; Winnenburg and Bodenreider 2014; WHO 2024)	All forms/strengths in one mechanism-of-action–based group
Coverage and structure	Incomplete therapeutic coverage	Exclusion of indicated drugs; incomplete or non-closed groups (Winnenburg and Bodenreider 2014)	Classify by mechanism of action or target without restriction to predefined therapeutic areas
	Vague/miscellaneous groups	“Other”/“various” catch-all groups lacking definition and logic (WHO 2024, 2018)	Mechanism-of-action– or target-based allocation; broad groups allowed but refinable
	Outdated structure and insufficient updates	Groups remain unchanged despite advances; innovative drugs placed in generic “X” classes instead of new specific levels (Sathyanarayana Rao and Andrade 2016; WHO 2018)	Maintain stable mechanism-of-action–based structure with regular updates to include new mechanisms without distorting analyses
Nomenclature and terminology	Imprecise or inconsistent naming	Group names not matching content; use of ambiguous “anti-” terms Winnenburg and Bodenreider 2014; Serafini et al. 2021	Use precise, neutral mechanism-of-action–based names (e.g., “mGPCR antagonists” instead of “antipsychotics”)
	Colloquial and effect-based terms	Use of everyday language (“blockers”, “stimulants”, “mimetics”) or broad functional labels (“therapy”) (Serafini et al. 2021; Robertson et al. 2019; Seifert and Alexander 2022)	Replace with standard pharmacologic or target-specific terminology (antagonists, agonists, inhibitors, etc.)
	Non-mechanistic or socially loaded labels	Chemical-only names without functional clarity, mechanism-diverse effect groups (“diuretics”), stigma-laden terms (“opioids”) (Seifert 2018a, 2018b)	Name by specific target/protein or pharmacologic mechanism (e.g., “MOR agonists”)

Moreover, the handling of combination products and overlapping therapeutic areas introduces additional ambiguity. The absence of consistent rules for such cases results in scattered or duplicated entries, with some drugs appearing in multiple codes while others are omitted (WHO 2025b; Günther et al. 2025). This inconsistency is not only problematic for data analysis, it also undermines the logic of the classification (Filippo Caraci et al. 2017; Sathyanarayana Rao and Andrade 2016). When using the ATC classification, it is frequently required to consult supplementary guidelines or apply subjective judgment to interpret or correct these inconsistencies, which contradicts the intended function as a standardized tool.

Another limitation is the chosen nomenclature. The terminology used within ATC codes is often outdated and too broad to provide a sufficient, precise and meaningful titling (Nelson et al. 2017; Winnenburg and Bodenreider 2014). Problematic terms such as “antiepileptics” or “antidiabetics” are used continuously, despite their incomplete, misleading and confusing effect (Seifert and Alexander 2022; Seifert 2018a; Filippo Caraci et al. 2017). The widespread use of “anti-” prefixes reinforces an indication-centric logic that is increasingly out of step with contemporary pharmacology. In many cases, group labels neither clarify mechanism nor reflect real-world therapeutic strategies, but instead perpetuate outdated or overly narrow understandings of drug function (Winnenburg and Bodenreider 2013; Serafini et al. 2021). This further distances the classification from both clinical and scientific relevance, and have a negative effect on patient adherence and treatment outcome (Ereso et al. 2024; Xiu-Juan et al. 2020).

Taken together, these structural, conceptual, and terminological issues expose a broader problem: the ATC system is not merely outdated, it is ill-suited to represent today’s pharmacotherapy. Its original logic is increasingly compromised by the realities of drug development, regulatory flexibility, and clinical practice (Robertson et al. 2019; Serafini et al. 2021; Nelson et al. 2017; Seifert 2018a; Günther et al. 2025). Therefore, relying on the ATC framework without critical evaluation may result in distorted views of pharmaceutical use, particularly in consumption studies.

## Future directions

### The need for a renewed classification and nomenclature

As it became evident, a reform of the current classification system is needed. Beside criticism, proposals need to be made for the development of a consistent, logical, sufficient, and comprehensive classification.

A mechanistically orientated nomenclature, based on pharmacological characteristics, appear to be the most rational and advantageous choice (Berlin et al. 2018; Kehrenberg and Bachmann 2022; Alvano and Zieher 2020; Filippo Caraci et al. 2017). Using this approach, drugs with similar characteristic are grouped together, being identified by their mechanism of action or molecular targets, grouping drugs with similar pharmacodynamic properties. It enables meaningful inferences about pharmacological effects and possible indications, without restricting drugs to a single therapeutic use or narrow clinical context (Alvano and Zieher 2020; Seifert 2018b). However, there are also limitations, including difficulties in handling of drugs with multi-targets (e.g., metformin) (Varrassi et al. 2020), differing selectivity (e.g., atropine) (Farinde 2025) or yet unknown mechanisms (e.g., paracetamol. Lithium, metamizole) (Davis 2020). In correspondence to this problem, a possible solution would be the classification in broader categories for not fully understood mechanisms, and subcategories for multi-targeting drugs (e.g., ibuprofen as a COX-1/2 inhibitor).

In general, the application of a mechanistically orientated drug classification system would result in a consistent and sufficient classification logic, where drugs and drug classes appear only once within the classification system (Alvano and Zieher 2020; Imming et al. 2004). Through this, the accuracy and interpretability of drug consumption data is improved, as well as misunderstanding and confusion is prevented (Talbe 9)). Furthermore, it enables the update and supplementation of the classification with innovational medicine, and does not contradict with the repurposing of drugs (Seifert and Schirmer 2021). While other options for drug classification are possible, e.g. by chemical structure, there emerges the disadvantage that drugs with a similar chemical structure do not necessarily share similar mechanisms of actions or effects (Wermuth et al. 2015; Kubinyi 2002), decreasing its value in clinical practice and education.

A terminology based on mechanistic rationale offers neutrality and precision (Seifert and Alexander 2022; Sanger and Andrews 2022). For example, using terms like mGPCR antagonists instead of traditional labels such as “antipsychotics”, avoids stigmatizing language that can affect both patients and healthcare professionals (Townsend et al. 2022; Chesaniuk et al. 2014). A neutral, descriptive nomenclature promotes more rational prescribing practices, improves therapeutic outcomes, and ultimately enhances patients’ quality of life while helping to reduce treatment costs for healthcare systems (Mekonnen et al. 2021; Goruntla et al. 2023). Table 8 provides an overview of how a mechanism-based nomenclature could address the issues identified in the ATC system.

### Progress towards a mechanistically-orientated classification

In recent years, a paradigm shift has occurred, moving from “one drug – one disease” to a multitargeted approach (Nogales et al. 2022; Yuan et al. 2025; Dasgupta 2025; Casas et al. 2019). As knowledge of medical conditions, underlying reasons and therapeutic strategies increases, previously hidden connections are becoming visible, enabling a new type of association and context (García Del Valle et al. 2019). This is evident not only pharmacology, but also in many other medical fields (Garzorz-Stark et al. 2025; Prieto Santamaría et al. 2021; Wadmann 2023; Berlin et al. 2018). These innovations in medicine must be reflected in reformed and adapted classification systems.

Progress has already been made through the adoption of mechanism-based nomenclature and classification systems. In Germany, especially within medical education, a mechanistically-oriented nomenclature is increasingly being established, supported by teaching resources such as “Basiswissen Pharmakologie” (Seifert 2018b), and by its integration into the IMPP’s official list of medicinal products for the second state examination in medicine since 2025 (IMPP 2024). Implementation in clinical medicine is also advancing, for instance in the annually published “Arzneiverordnungsreport” (Drug Prescription Report, Germany) (Ludwig et al. 2025), and through the German Society for Internal Medicine (DGIM), which applies it in their essential medicines list (DGIM 2025). Nevertheless, progress remains gradual, and much work is still required to achieve a consistent and rational nomenclature (Bindel and Seifert 2025c).

## Supplementary Information

Below is the link to the electronic supplementary material.

Supplementary file1 (DOCX 63.7 KB)

## Data Availability

All source data for this study are available upon reasonable request from the authors.

## Abbreviations

ACE inhibitor: Angiotensin-converting enzyme inhibitor
AChEI: Acetylcholinesterase inhibitor
Alpha-2AR antagonist: Alpha-2 adrenergic receptor antagonist
ARB: Angiotensin receptor blocker
ASS: Acetylic salicylic acid
ATC: Anatomical therapeutic chemical classification system (drug classification system, WHO)
BAN: British approved name
CLL: Chronic lymphocytic leukemia
COX inhibitor: Cyclooxygenase inhibitor
DDD: Defined daily dose
DGIM: German Society for Internal Medicine
EGFR inhibitor: Epidermal growth factor receptor inhibitor
GORD: Gastro-oesophageal reflux disease
HER2 inhibitor: Human epidermal growth factor 2 inhibitor
IMPP: “Institut für medizinische und pharmazeutische Prüfungsfragen” (Institute for Medical and Pharmaceutical Examination Questions, Germany)
INN: International nonproprietary name
IUPHAR: International union of basic and clinical pharmacology
JAK inhibitor: Janus kinase inhibitor
MOR agonist: µ-Opioid receptor agonist
mTOR inhibitor: Mammalian target of rapamycin kinase inhibitor
MxR agonist: Muscarinic receptor agonist
NaSSA: Noradrenergic and specific serotonergic enhancer
NbN: Neuroscience-based nomenclature
NCC: Na + /Cl- cotransporter
NE/5-HT enhancer: Norepinephrine and serotonin enhancer
NKCC: Na + /K + /2Cl- cotransporter
NKLM: “Nationaler kompetenzbasierter Lernzielkatalog Medizin” (National competence-based learning objectives catalogue for medicine, Germany)
NMDA antagonist: N-methyl-D-aspartate antagonist
NSAID: Non-steroidal anti-inflammatory drug (COX inhibitor)
PAH: Pulmonary arterial hypertension
PPI: Proton pump inhibitor
SLGT-1/2 inhibitor: Sodium-glucose cotransporter 1/2 inhibitor
SSRI: Selective serotonin reuptake inhibitors
TYK2 inhibitor: Tyrosine kinase 2 inhibitor
USAN: United States approved name
WHO: World Health Organization
