# Supplementary material for: Problems associated with the ATC system of drug classification
Source: Naunyn Schmiedebergs Arch Pharmacol. 2025 Dec 1;399(5):6911–38. doi: 10.1007/s00210-025-04833-1 (PMC13053441; doi:10.1007/s00210-025-04833-1)
Supplement: Supplementary file 1 — Supplementary file1 (DOCX 63.7 KB) [file 210_2025_4833_MOESM1_ESM.docx]

**Problems associated with the ATC system of drug classification –**

**Supplement**

**Lilly Josephine Bindel and Roland Seifert**

**Institute of Pharmacology
Hannover Medical School
Carl-Neuberg-Str. 1
D-30625 Hannover, Germany
Corresponding author: Roland Seifert
seifert.roland@mh-hannover.de**

***Table 2:*** *Summary of ATC subgroup analysis across all main groups, presenting ATC codes and group titles together with key findings on structural inconsistencies, combination product handling, and overlaps.*

| **ATC code** | **group title** | **classification consistency issues** | | **combination handling issues** | | | **key redundancy and overlap (therapeutic and pharmacologic groups)** |
| --- | --- | --- | --- | --- | --- | --- | --- |
|  |  | ***mixed classification criteria in subgroups (level 3);***  ***other notable findings***  *(WHO 2024a)* | ***exluded relevant drugs***  *(WHO 2024b)* | ***combinations of listed plain drugs placed outside group***  *(WHO 2024b)* | ***combinations with other drugs included in plain substance groups***  *(WHO 2024a, 2024b)* | ***separate combination category***  *(WHO 2024a)* |  |
| **A** | **Alimentary tract and metabolism** | | | | | | |
| A01 | stomatological preparations | therapeutic, pharmacologic, miscellaneous | exclusion of throat infections or both mouth and throat indication (R02), exclusion of local anesthetics (N01B, R02A), exclusion of corticosteroids for local use (D), exclusion of local antibacterials (D), exclusion of colecalciferol and sodium fluoride for caries prophylaxis (A11CC), missing combinations | stomatological preparations + local anesthetics (N01B, R02AD) | corticosteroids/other drugs for local oral treatment + local anesthetics (A01AC/AD), antibacterials + anti-inflammatoric drugs (A01AB) | - | antiinfectives (A07, B05, D, G01, J, S), corticosteriods (A, C, D, G, H, M, N, R, S), anesthetics (A, C, D, N, R, S) topical use, systemic use  (Addy and Fugit 1989) |
| A02 | drugs for acid related disorders | chemic, therapeutic, miscellaneous | exclusion of plain antiflatulents (A03AX), exclusion of other bismuth salts than ranitidine bismuth citrate (A02BX), exclusion of parenteral formulation of antacids with sodium bicarbonate (B05BB), missing combinations | gastro-oesophageal reflux disease (GORD) drugs + cyclooxygenase (COX)-inhibitors (M01A), antacids + sodium bicarbonate in double-contrast radiography (V07AY), antacids + COX-inhibitors (M01A) | GORD + H2-receptor antagonists (A02B), proton pump inhibitors + domperidone (A02BC), alginic acid + antacids (A02BX), domperidone + proton pump inhibitor (A02BC) | A02A(D-X), A02BD | COX-inhibitors, antibacterials, drugs for functional gastrointestinal disorders (A03), haemostatic drugs (B02), prostaglandins (A02, C01, G02), misoprostol (A02BB, G02AD)  (Savarino et al. 2017, 2021) |
| A03 | drugs for functional gastrointestinal disorders | therapeutic, pharmacologic, miscellaneous | exclusion of drugs for constipation (A06), antispasmodics for the urogenital tractus (G04BD), missing combinations | antispasmodics + psycholeptics (partly N05, partly A03); antispasmodics + analgesics (partly N02, partly A03) | papaverine + sterculia (A03AD), silicones + antispasmodics (A03AX), silicones + antacids (A02AF), silicones + antipropulsives (A02AF), antispasmodics + psycholeptics + analgesics (A03EA, A03C) | A03C, A03D, A03E | analgesics (A03, C03, N02, S02), spasmolytic drugs (A02, A03, N02), psycholeptics (A, N, R), urogenital tractus (G), constipation (A06), trospium (G04BD, A03AB, A03BA), papaverine (A03AD, G04BE), A03C and A03D, propulsives (A03AA, A03FA, N05AL), histamine receptor antagonists (D04, M03, N04, R06); nervous system (N)  (Drossman et al. 2018 ; Singh et al. 2022) |
| A04 | antiemetics and antinauseants | pharmacologic, miscellaneous | exclusion of histamine receptor antagonist (R06), exclusion of droperidol (N05AD), metoclopramide (A03FA), missing combinations | antiemetics/antinauseants + analgesics (N02) | palonosetron + other drugs (A04AA) | - | histamine receptor antagonist (D04, R06, M03, N04), antivertigo preparations (N07), mGPCR antagonists (N05)  (Herrstedt 2018; Athavale et al. 2020; Fonte et al. 2015) |
| ***Table 2 (continued)*** | | | | | | | |
| ***ATC code*** | ***group title*** | ***mixed classification criteria, other findings*** | ***excluded relevant drugs*** | ***combinations placed outside group*** | ***combinations with other drugs included*** | ***separate category*** | ***key redundancy and overlap*** |
| A05 | bile and liver therapy | therapeutic, chemic | exclusion of thioctic acid (A16AX), missing combinations | laxatives + centrally acting antiobesity drugs (A08A), contact laxatives + bulk producing laxatives (A06AC) | bile acids + combinations (A05AA) | A05C | drugs against obesity (A08), diabetes (A10), lipid-modifying agents (C10), antihypertensive drugs (C02), vitamins (A11, B), immunomodulating agents (L)  (Chiang 2025; Ali et al. 2023; Gilgenkrantz et al. 2025) |
| A06 | drugs for constipation | pharmacologic, chemic, miscellaneous | exclusion of combinations with centrally acting antiobesity drugs (A08A), missing combinations | magnesium + albumin tannate (A07XA) | softeners/emollients + contact laxatives (A06AB), softeners/emollients + liquid parrafins (A06AA), contact laxatives + osmotically acting laxatives (A06AB) | - | -  (Luthra et al. 2019; Rao and Brenner 2021) |
| A07 | antidiarrheals, intestinal, anti-inflammatory/  antiinfective agents | therapeutic, pharmacologic, chemic, miscellaneous | exclusion of systemic antiinfectives, exclusions parenteral formulations of vancomycin (J01XA), exclusion of telotristat (A16AX) | - | neomycin + other antibacterials (A07AA), adsorbents + antiinfectives (A07A), adsorbents + albumin tannate (A07XA), diphenoxylate + atropine (A07DA), opium + elladonna/bismuth subgallate/albumin (A07DA), MOR-agonists + aluminium hydroxide/belladonna alkaloids/albumin (A07DA), antipropulsives + antiflatulents (A07DA), antidiarrheals + pectin/magnesium peroxide (A07XA) | - | systemic vs local antiinfectives, antiinflammatories, antiparasitics/ insecticides/repellents (P), A07C and V06, antibacterials with sulfonamides, imidazole derivatives and other antiinfectives (A07AA-AX), bismuth preparations and GORD (A07BB, A02BX), MOR agonists (A07DA, N02AA), antiinflammatoric drugs, cromoglicic acid (A07, D11, R, S01), aminosalicyclic acid (J04), bile and liver therapy (A05)  (Lee 2015; Khan et al. 2011; Cai et al. 2021) |
| A08 | antiobestiy preparations, excl. diet products | pharmacologic, miscellaneous | exclusion of low-energy diets (V06AA), exclusion of amfetamine (N06B), exclusion of fenfluramine (N03AX), liraglutide, semaglutide, tirzepatide (A10B) | - | - | - | low-energy diets (V06AA), diabetes (A10), psychoanaleptics (N06), bile and liver therapy (A05)  (Chakhtoura et al. 2023; Son and Kim 2020) |
| ***Table 2 (continued)*** | | | | | | | |
| ***ATC code*** | ***group title*** | ***mixed classification criteria, other findings*** | ***excluded relevant drugs*** | ***combinations placed outside group*** | ***combinations with other drugs included*** | ***separate category*** | **key redundancy and overlap** |
| A09 | digestives, incl. enzymes | - | exclusion of enzymes for inflammatory conditions (M09AB), exclusion of cholagogues (A05), missing combinations | digestive enzymes + other drugs if main indication is not digestion disorder | digestive enzymes + other drugs if main indication is digestion disorder (A09AA) | A09AC | enzymes (B06AA), proteolytic enzymes (D03BA), bile and liver therapy (A05), inflammatory conditions (L)  (Baldo 2015; Kadaj-Lipka et al. 2025) |
| A10 | drugs used in diabetes | pharmacologic, chemic, miscellaneous | missing combinations | nateglinide + thioctic acid (A10BX) | SGLT1- + SGLT2- inhibitors (A10BK) | A10AD, A10BD | thioctic acid (A16), bromocriptine (G02CB, A10BX), antiobesity drugs (A08)  (Dara et al. 2025; Salehi et al. 2019 ; Haddad et al. 2023) |
| A11 | vitamins | chemic, therapeutic, miscellaneous | exclusion of Vitamin B12 (B03), exclusion of Vitamin K (B02), exclusion of vitamins as i.v. solution additives (B05XC), exclusion of iron with more than 30mg Fe2+ (B03A), missing combinations | cholecalciferol + sodium fluoride (A11CC), ascorbic acid + analgesics (N02B), vitamins + folic acid if folic acid deficiency is main indication (B03BB), ascorbic acid + calcium if indicated for calcium deficiency or osteoporosis (A12AX) | multivitamins + minerals (A11AA), multivitamins + mineral + other drugs (A11AB), vitamin B + minerals (A11EC), vitamin B + anabolic steroids (A11ED) | A11A, A11CB, A11CC, A11D, A11E, A11G, A11J | antianemic drugs/ iron (B03), antihemorrhagics (B02), blood substitutes (B05), mineral supplements (A12), calcium homeostasis (H05), hormones (H), calciferol, paricalcitol and doxercalciferol in other anti-parathyroid agents (H05BX), prophylaxis of rickets and caries (A11), tonics (A13)  (Gombart et al. 2020; Alberts et al. 2025 ; Lai et al. 2025) |
| A12 | mineral supplements | chemic, miscellaneous | exclusion of parenteral solutions of electrolytes (B05B/X), exclusion of magnesium carbonate (A02AA), exclusion of fluoride in caries prophylaxis (A01AA), missing combinations | calcium acetate + magnesium carbonate (V03AE), calcium carbonate + antacids (A02AC), calcium + biphosphonates (M05BB), potassium + diuretics (C03) | calcium + vitamin D (A12AX), calcium + fluoride (A12CD), potassium + other drugs (A12BA) | A12AX | antacids (A02A), hyperphosphatemia (V03AE), vitamins (A11), blood substitutes (B05), bone structure and mineralization (M05B), diuretics (C03)  (Liu et al. 2015; Fishman et al. 2000) |
| A13 | tonics | - | catch-all group when requirements are not fulfilled for classification as iron or vitamin preparations |  |  |  | vitamins (A11), mineral supplements (A12), general nutrients (V06) |
| A14 | anabolic agents for systemic use | chemic, miscellaneous | exclusion of anabolic steroids for cancer therapy (L), exclusion of prasterone for vaginal use (G03X) | - | - | - | antineoplastic and immunomodulating agents (L), gynecologics (G)  (Suvannasankha and Chirgwin 2014; Inderjeeth and Inderjeeth 2024; Marshall-Gradisnik et al. 2008) |
| A15 | appetite stimulants | - | exclusion of cyproheptadine (R06AX), exclusion of pizotifen (N02CX), exclusion of megestrol (L02AB) | - | - | - | respiratory system/ histamine receptor antagonists (R06AX), analgesics (N02CX), antineoplastic drugs/ endocrine therapy (L02AB), NE/5-HT antagonists  (Steiner et al. 2023; Harrison et al. 2019 ; Chinuck et al. 2014) |
| A16 | other alimentary tract and metabolism products | chemic, miscellaneous | catch-all group for A, missing combinations | tryptophan + oxitriptan (N06A) |  |  | other therapeutic products (V03), general nutrients (V06), enzymes (A09, B, C, D03, M09), A01-A15 |
| ***Table 2 (continued)*** | | | | | | | |
| ***ATC code*** | ***group title*** | ***mixed classification criteria, other findings*** | ***excluded relevant drugs*** | ***combinations placed outside group*** | ***combinations with other drugs included*** | ***separate category*** | ***key redundancy and overlap*** |
| **B** | **blood and blood forming organs** | | | | | | |
| B01 | antithrombotic agents | therapeutic, pharmacologic, miscellaneous; exception principle of only one code for each rout of administration for acetylsalicylic acid as a platelet aggregation inhibitor | sulfinpyrazone (M04AB), alprostadil (C01EA, G04BE), missing combinations | acetylsalicylic acid + statins (C10BX), acetylsalicylic acid + beta-adrenergic receptor antagonists (C07FX) | acetylsalicylic acid + rivaroxaban (B01AF) | - | vitamins (A11, B02, V), platelet aggregation inhibitors (C01/07/10), acetylsalicylic acid (N02BA, B01AC), prostaglandins (B01AC, C02KX, G04BE), enzymes (A09, B, C, D03, M09)  (Galanti et al. 2024; Gupta et al. 2022) |
| B02 | anti-hemorrhagics | therapeutic, chemic, miscellaneous | exclusion of fibrinogen for other application that systemic, exclusion of local haemostatics (A01AD), exclusion of epinephrine injection (C01C), exclusion of tissue adhesives (V03AK) | - | antifibrinolytics + vitamin K (B02A), fibrinogen/aprotinin/  thrombin + collagen (B02BC) | - | vitamins (A11, B01, V), haemostatics (A01AD, C01C, V03AK, B02BC)  (Jamali et al. 2024; Harter et al. 2015) |
| B03 | antianemic preparations | chemic, miscellaneous; classification of hydroxocobalamin for indication of neuralgia in B03BA | exclusion of iron preparations with less than 30mg Fe (A11, A13), exclusion of folic acid for diagnostic use (V04CX), exclusion of folinates for antidotes (V03A), exclusion of glutamine for treatment of sickle cell disease (A16AA), exclusion of cell-and gene-therapy products for Fanconi anaemia and sickle cell disease (B03AX) | - | iron + stabilizing agents/laxatives/  folic acid (B03A) | B03AD, B03AE | vitamins (A11, B, V)  (Bereda 2022) |
| B05 | blood substitutes and perfusion solutions | therapeutic, pharmacologic, chemic, miscellaneous | exclusion of blood transfusion (V07AC), exclusion of irrigation solution for non-therapeutic use (V07AB), missing combinations | parenteral nutrition + electrolyte + glucose (B05BB) | parenteral nutrition + electrolytes (B05BA) | - | solvents and diluting agents (V07AB), blood transfusion (V07AC), antiinfectives, mineral supplements (A12), vitamins (A11)  (Hoorn 2017) |
| B06 | other hematological agents | therapeutic, pharmacologic, chemic, miscellaneous; catch-all group for B | exclusion of givosiran from heme products (A16AX), exclusion of glutamine for sickle cell disease (A16AA) | - | - | - | all other non-therapeutic products (V07A), heparins (B01AB), enzymes (A09, B01, D03), other surgical aids (S01) |
| ***Table 2 (continued)*** | | | | | | | |
| ***ATC code*** | ***group title*** | ***mixed classification criteria, other findings*** | ***excluded relevant drugs*** | ***combinations placed outside group*** | ***combinations with other drugs included*** | ***separate category*** | ***key redundancy and overlap*** |
| **C** | **cardiovascular system** | | | | | | |
| C01 | cardiac therapy | therapeutic, miscellaneous; variation in Vaughan Williams classification of antiarrhythmics depending on literature | exclusion of antiarrhythmics class 2 (C07) and class 4 (C08), exclusion of lidocaine (N01BB), exclusion of phenytoin (N03), exclusion of sotalol (C07AA), exclusion of dihydroergotamine (N02CA), exclusion of oral products of ephedrine (R03CA), exclusion of amyl nitrite (V03AB), missing combinations | cardiac glycosides + antihypertensives/beta-adrenergic receptor antagonists/calcium channel inhibitors/ ACE inhibitors (C02/07/08/09), antiarrhythmics + quinidine/ verapamil (C08DA), cardiac stimulants + peripheral vasodilators (C04), vasodilators + cardiac glycosides/ rauwolfia alkaloids/ beta-adrenergic receptor antagonists/ calcium channel inhibitors (C01A, C02A, C07, C08), ivabradine + beta-adrenergic receptor antagonists (C07FX) | cardiac glycosides + drugs of C01D/E in C01A, digitalis glycosides + diuretics (C01AA), etilefrine + dihydroergotamine (C01CA), isosorbide dinitrate + hydralazine (C01DA), nitrate preparations + psycholeptics in (C01DA) | C01EX | vasodilators/ischemic heart disease (C02, C03, C04, C07, C08, C09), antiarrhythmics (C07, C08), stimulants (R07), migraine (N02), phosphodiesterase inhibitors (R03D), antidotes (V03AB), prostaglandins (G04BE), adenosine, cardiovascular drugs (C02-C09)  (Van Gelder et al. 2024; McDonagh et al. 2021) |
| C02 | antihypertensives | therapeutic, pharmacologic, miscellaneous | exclusion of alpha- and beta-adrenergic receptor antagonists (C07AG), exclusion of alfuzosin and terazosin (G04CA), exclusion of oral preparations of diazoxide (V03AH), exclusion of dermatological preparations of minoxidil (D11AX), exclusion of phosphodiesterase inhibitors and prostaglandins for PAH (G04BE, B01AC), missing combinations | rauwolfia alkaloids +beta-adrenergic receptor antagonists (C07F), isosorbide dinitrate + hydralazine (C01DA), diuretics + calcium channel inhibitors/ ACE inhibitors /angiotensin 2 receptor blockers (C07, C08, C09BA, C09DA) | antihypertensives + diuretics (C02L) | C02L, C02N | antihypertensive drugs (C02, C03, C07, C08, C09), clonidin and guanfacine in ADHD and migraine (N02, N06), calcium channel inhibitors (C08), diazoxide (V03), minoxidil (D11), pulmonary arterial hypertension (G04BE, B01AC),  (Shan et al. 2022; Carcel et al. 2023) |
| C03 | diuretics | therapeutic, pharmacologic, miscellaneous | many combinations with diuretics classified in other groups (eg. C01AA, C02L, C08B), missing combinations | diuretics + digitalis glycosides (C01AA), diuretics + antihypertensives (C02L), diuretics + beta-adrenergic receptor antagonists (C07B-D), diuretics + calcium channel inhibitors (C08), diuretics + drugs acting on the renin angiotensin system (C09B/D) | sulfonamides + psycholeptics (C03BK) | C03CB, C03E | potassium (A12), xanthines (R03), GCR agonists (C03, H02), finerenone and chronic kidney disease, cardiovascular conditions like hyptertension and heart insufficiency (C), diabetes (A10), ophthalmology (S01)  (Kehrenberg and Bachmann 2022; Roush et al. 2014; Tang et al. 2022) |
| ***Table 2 (continued)*** | | | | | | | |
| ***ATC code*** | ***group title*** | ***mixed classification criteria, other findings*** | ***excluded relevant drugs*** | ***combinations placed outside group*** | ***combinations with other drugs included*** | **separate category** | **key redundancy and overlap** |
| C04 | peripheral vasolidators | chemic, miscellaneous | exclusion of nicotinic acid in high strength (C10AD), exclusion of papaverine preparations (A03AD, G04BE), exclusion of prostaglandins for PAH or arteriosclerosis obliterans (B01AC), missing combinations | peripheral vasodilators + antihypertensives (C02), combinations of vasodilators for cardiac diseases (C01DA), cinnarizine + dihydroergocryptine (N07CA), ergot alkaloids + calcium channel inhibitors (C08CA) | purine derivatives + nicotinic acid (C04AD), ergot alkaloids + other peripheral vasodilators (C04AE) | - | cholesterol reducer (C10), ergot alkaloids (G02AB, N02CA), enzymes (A09, A16, B01, B06, C04, D03, M09), antivertigo drugs (N07)  (Hairi and Patel 2023) |
| C05 | vasoprotectives | therapeutic, miscellaneous; included/excluded application forms inconsistent | exclusion of systemic antiinfectives J), corticosteroids (A, C, D, G, H, S), anesthetics (N01), exclusion of zinc bandages (D09A), missing combinations | heparin + diclofenac for topical use (M02AA) | local corticosteroids + antiinfectives/local anesthetics (C05AA), heparin + dexpanthenol/allantoin (C05BA), heparinoids + calcium dobesilate (C05BX), bioflavonoids + other capillary stabilizing drugs (C05CA) | - | corticosteroids (A, C, D, G, H, S), antibacterials (A, C, D, G, J, L, R, S), anesthetics (D04, N01), medicated dressings (D09), beperminogene perplasmid for chronic arterial occlusive disease (B, C, D)  (Gohel and Davies 2009; Gontijo et al. 2017; Hardung et al. 2021) |
| C07 | beta blocking agents | therapeutic, pharmacologic, miscellaneous | missing combinations | beta-adrenergic receptor antagonists + ACE inhibitors (C09BX), beta-adrenergic receptor antagonists + ARBs (C09DX), beta-adrenergic receptor antagonists + statins (C10BX) | beta-adrenergic receptor antagonists + thiazides/diuretics/  others (C07) | C07B, C07C, C07D, C07E, C07F | blood and blood forming organs (B), cardiovascular conditions (C)  (Diagonu et al. 2019) |
| C08 | calcium channel blockers | therapeutic, pharmacologic | missing combinations | calcium channel inhibitors + ergot alkaloids (C04AE), calcium channel inhibitors + ACE inhibitors (C09BB), calcium channel inhibitors + beta-adrenergic receptor antagonists (C07BF), calcium channel inhibitors + statins (C10BX), amlodipine + atorvastatin (C10BX) | nifedipine + ergot alkaloids (C08CA), verapamil + quinidine (C08DA), calcium channel inhibitors + diuretics (C08GA) | C08G | blood and blood forming organs (B), diuretics (C03), cardiovascular conditions (C), nervous system (N) (Sica 2007; Lee 2023; Manzar et al. 2025) |
| ***Table 2 (continued)*** | | | | | | | |
| ***ATC code*** | ***group title*** | ***mixed classification criteria, other findings*** | ***excluded relevant drugs*** | ***combinations placed outside group*** | ***combinations with other drugs included*** | ***separate category*** | ***key redundancy and overlap*** |
| C09 | agents acting on the renin-angiotensin system | pharmacologic, miscellaneous | missing combinations | ACE inhibitors + statins + acetylsalicylic acid (C10BX) | ACE inhibitors + diuretics (C09BA), ACE inhibitors + calcium channel inhibitors (C10BB), ACE inhibitors + other drugs (C09BX) | C09B | blood and blood forming organs (B), cardiovascular system (C), antineoplastic agents (L)  (Zaman et al. 2002; Pawlonka et al. 2024) |
| C10 | lipid modifying agents | - | exclusion of patethine (A11HA), exclusion of nicotinic acid in low strengths (C04A), exclusion of sulodexide (B01AB), missing combinations | blood glucose-lowering drugs + lipid modifying agents (A10B) | atorvastatin + amlodipine (C10BX), nicotinic acid + laropiprant (C10AD), lipid modifying drugs + ACE inhibitors /ARBs/diuretics/ beta-adrenergic receptor antagonists/ calcium channel inhibitors (C10BX) | C10B | vitamins (A11, B, V06), bile and liver therapy (A05), nicotinic acid and peripheral vasodilators (C04A), diabetes (A10), blood and blood forming organs (B)  (Simons 2019) |
| **D** | **dermatologicals** | | | | | | |
| D01 | antifungals for dermatological use | therapeutic, anatomic | exclusion of topical drugs for gynaecological infections (G01), exclusion of drugs for local fungal infections in the mouth (A10AB), exclusion of topical metronidazole for rosacea (D06BX) | - | antibacterials + antiseptics (D01AA), imidazole/triazole + corticosteroids (D01AC), antifungals + salicylic acid (D01AE) | - | antimycotics (J02A), gynaecological infections (G), local infections in the mouth (A01), antibacterials (J, D06A, ...), imidazoles, corticosteroids (D07, H02, ...), rosacea (D06), quinolone derivatives (D08)  (Carmo et al. 2023; Hoenigl et al. 2024) |
| D02 | emollients and protectives | catch-all group for all types of emollients and protectives with no specific therapeutic effect or use and non-classified preparations of medicated dressings (D09) | exclusion of non-medicated adhesive plasters, surgical tapes and others (V07AA), exclusion of medicated shampoos with salicylic acid (D11AC), missing combinations | salicylic acid + corticosteroids (D07X), salicylic acid + topical products for joint and muscular pain (M02AC) | salicylic acid + acne drugs/others (D02AF) | - | medicated dressings, cicatrizants (D), various (V07), salicylic acid, boric acid products (D08AD), musculo-skeletal system (M), dermatological diseases (D)  (Nankervis et al. 2016; Jacobi et al. 2015) |
| D03 | preparations for treatment of wounds and ulcers | catch-up group D03A for topical vitamin preparations that are not-classifiable in other groups | exclusion of topical products with glyceryl trinitrate/isosorbide dinitrate for anal fissures (C05AE), exclusion of protective ointments (D02A) | - | vitamin A + chlorhexidine (D03AA), dextranomer powders + antiseptics (D03AX) | - | protective ointments (D02A), antibacterials (D06), antiseptics (D08), antiinfectives (D06, J), medicated dressings (D09), vitamins (A, B, V), enzymes (A, B, C, M)  (Dhivya et al. 2015; Ffrench et al. 2023) |
| ***Table 2 (continued)*** | | | | | | | |
| ***ATC code*** | ***group title*** | ***mixed classification criteria, other findings*** | ***excluded relevant drugs*** | ***combinations placed outside group*** | ***combinations with other drugs included*** | ***separate category*** | ***key redundancy and overlap*** |
| D04 | antipruritics, incl. antihistamines, anesthetics, etc. | therapeutic, miscellaneous; only topical use, ATC level 5 plain drugs and combinations often not differentiated (e.g. D04AA, D04AB) | antipruritics listed in other classifications (e.g. D02, D08, M02, V03AX), incomplete catch-all group "other antipruritics" (D04AX), exclusion of nafurafine and difelikefalin (V03AX), missing combinations | histamine receptor antagonist + corticosteroids (D07) | histamine receptor antagonist + anesthetics (D04AB) | - | corticosteroids in dermatological preparations (D07), haemorrhoids and anal fissures (C05A), local anesthetics (N01B), emollients and protectives (D02), antiseptics and disinfectants (D08), topics for joint and muscular pain (M02)  (Elmariah and Lerner 2012) |
| D05 | antipsoriatics | plain corticosteriods excluded while corticosteriods in combination classified in D05AX | exclusion of corticosteroids for topical use (D07), exclusion of antineoplastic and immunomodulating agents although indicated for severe psoriasis (L04), missing combinations | tars + corticosteroids (D07) | vitamin D analogues + corticosteroids (D05AX) | - | corticosteroids (D07), antineoplastic and immunomodulating agents (L), retinoids (D10, D11)  (Afifi et al. 2005; Maniyan et al. 2020) |
| D06 | antibiotics and chemo-therapeutics for dermatological use | mixture of plain and combination preparations in oxytetracycline/neomycin/bacitracin unter a single ATC code (D06AA, D06AX); D06BX summarizes chemotherapeutics for different skin disorders that cannot be classified in existing groups | exclusion of antibacteials for dermatological use with antimycotic properties, combinations with chemotherapeutics, combinations with corticosteriods, or antiinfectives for treatment of acne (D01A, D06C, D07C, D10AF), exclusion of mucoadhesive formulations of aciclovir (J05AB), missing combinations | antiinfectives + corticosteroids (D07) | oxytetracycline/neomycin/bacitracin + other antibacterials (D06AA) | - | antiinfectives (D01A, D06C, D07C, D10), other dermatologicals (D11AX), antineoplastic agents (L01), dermatologic diseases (D), antiseptic drugs (D08)  (Dallo et al. 2023) |
| D07 | corticosteroids, dermatological preparations | pharmacologic, therapeutic, miscellaneous | exclusion of corticosteroids for local oral treatment (A01AC), exclusion of anti-acne preparations (D10A), exclusion of ophthalmological and otological use (S), missing combinations | corticosteroids + antifungals (D01A), corticosteroids + antihemmorhoidals (C05AA) | corticosteroids + antiseptics (D07B), corticosteroids + antiseptics + salicylic acid (D07X), corticosteroids + antibacterials (D07C), corticosteroids + other drugs (D07X) | D07B, D07C, D07X | local oral treatment (A01), intiinfectives (D01), acne (D10), hemorrhoidals (C05AA), sensory organs (S), salicylic acid (A, D, M, ...)  (Das and Panda 2017; Lata et al. 2020) |
| D08 | antiseptics and disinfectants | pharmacologic, chemic, miscellaneous | catch-all group for dermatological antiinfective drugs which are not classified on other groups (D01, D03A, D06, D07X, D07B, D09A, D10A, D11AC, P03A, D06BB) | - | chloroquinaldol + clioquinol (D08AH), mercurial + silver compounds (D08AK) | - | antifungals (D01), cicatrizants (D03), antibacterials (D06), corticosteroids (D07), medicated dressings and shampoos (D09, D11), acne (D10A), extoparasiticides (P03A), antivirals (D06), creams and lubricants (V07)  (Babalska et al. 2021; Williamson et al. 2017) |
| ***Table 2 (continued)*** | | | | | | | |
| ***ATC code*** | ***group title*** | ***mixed classification criteria, other findings*** | ***excluded relevant drugs*** | ***combinations placed outside group*** | ***combinations with other drugs included*** | ***separate category*** | ***key redundancy and overlap*** |
| D09 | medicated dressings | therapeutic, chemic | exclusion of liquid wound protectives (D02AD), exclusion of local haemostatics (B02BC), exclusion of hyaluronic acid preparations (D03AX), exclusion of products with cadexomer iodine (D03AX), missing combinations | medicated dressings + antiinfectives (D03AX, D08AG) | medicated dressings + antiinfectives (D09AA) | - | liquid plasters (D02AD), local hemostatics (B02BC), other cicatrizants (D03AX), antiinfectives (D03AX, D08AG)  (Ghomi et al. 2019; Bhojar et al. 2023) |
| D10 | anti-acne preparations | pharmacologic, chemic, miscellaneous | exclusion of tetracyclines and erythromycin (J), exclusion of sex hormones (G03) | - | corticosteroids + retinoids (D10AD), corticosteroids + peroxides (D10AE) sulfur + resorcinol (D10AB), retinoids + antibacterials (D10AD) | D10AA | antiinfectives (D06, J), corticosteriods (D07), hormones (G03, H) |
| D11 | other dermatological preparations | therapeutic, pharmacologic, miscellaneous; catch-all group for dermatological preparations that do not fit in D01-D10 | exclusion of insect repellents (P03B), exclusion of glycopyronium bromide for systemic use (A03AB), exclusion of shampoos containing imidazoles and coal tar (D01AC, D05AA), exclusion of podophyllotoxin/podophyllin for genital warts (D06BB), exclusion of corticosteroids (D07) | - | - | - | glycopyrronium (A03AB), medicated shampoos (D01, D05), genital warts (D06), seborrheic keratosis and warts (D11AX), D01-D10  (Mohsin et al. 2022) |
| **G** | **genito urinary system and sex hormones** | | | | | | |
| G01 | gynecological antiinfectives and antiseptics |  | exclusion of antivirals for topical use (D06), exclusion of parenteral imidazole formulations (J01XD), missing combinations | antinfectives/antiseptics + corticosteroids (G01B) | nystatin + nifuratel (G01AA), econazole + benzydamine (G01AF) | G01B | antiinfectives (J, D, P), dermatologicals (D)  (Giouleka et al. 2025) |
| G02 | other gynecologicals | therapeutic, pharmacologic, miscellaneous | exclusion of analgesics used in dysmenorrhea (N02B, M01A), exclusion of oxytocin (H01B), exclusion of contraceptives for systemic use (G03A), missing combinations | econazole + benzydamine (G01AF) | - | G02AC | analgesics and antiinflammatory drugs (N02B, M01A), hormones (H01), ergot alkaloids (C04A, N02C), prostaglandins (A02), contraceptives (G03), adrenergic drugs (R03), prolactin inhibitors (N04, N02C) |
| G03 | sex hormones and modulators of the genital system | therapeutic, chemic, miscellaneous; tibolone classified in "other estrogens" although differing structure (G03CX) | exclusion of sex hormones only used in the treatment of neoplastic diseases (L), exclusion of intravaginal and intrauterine hormone-containing devices (G02B), exclusion of norethandrolone (A14A), exclusion of systemic formulations of prasterone (A14AA), exclusion of finasteride (G04CB) | - | cyproterone + estrogen (G03HB), mifepristone + misoprostol (G03XB) | G03AA, G03CC, G03E, G03F, G03HB | hormones (H), antineoplastic and immunomodulating agents (L), urogenital tract (G), anabolic steroids (A14A), prasterone (A14AA), nervous system (N)  (Harper-Harrison et al. 2024; McEven and Milner 2017) |
| G04 | urologicals | therapeutic, pharmacologic, miscellaneous | exclusion of potassium (A12BA), exclusion of gastrointestinal antispasmodics (A03), exclusion of alpha-adrenoreceptor blocking agents indicated for both urinary obstruction and hypertension (C02CA, eg doxazosin), missing combinations | trospium + analgesics (A03DA), phosphodiesterase inhibitors + ambrisentan/macitentan (C02KX, B01AC), phenazopyridine + sulfonamides (J01EB/D) | - | G04BE | antiinfectives (G01, J), mineral supplements (A12), antispasmodics (A03), alprostadil (C01EA), phsophodiesterase inhibitors (C02, B01), local anesthetics (N01B), alpha-adrenoreceptor blocking drugs (C02)  (Liu et al. 2011) |
| ***Table 2 (continued)*** | | | | | | | |
| ***ATC code*** | ***group title*** | ***mixed classification criteria, other findings*** | ***excluded relevant drugs*** | ***combinations placed outside group*** | ***combinations with other drugs included*** | ***separate category*** | ***key redundancy and overlap*** |
| **H** | **systemic hormonal preparations, excl. sex hormones and insulins** | | | | | | |
| H01 | pituitary and hypothalamic hormones and analogues | mecasermin (insulin like growth factor) classified in somatropin (H01AC) | exclusion of diagnostic purposes (V04), exclusion of beserelin, goserelin, histrelin, leuprorelin, triptorelin (L02AE), missing combinations | oxytocin + ergot alkaloids (G02A) | - | - | hormones (A, C, G, R, L), thyrotropin (V04), oxytocin (G02A), gonadotropin-releasing hormones (L02), antineoplastic therapy (L01, L02), nervous system (N)  (Iglesias 2024; Soria et al. 2018; Raja et al. 2022) |
| H02 | corticosteriods for systemic use | exception of rule that all systemic corticosteriods are included (M01BA) | exclusion for many application forms (A01AC, A07AE, D07, D10AA, G01B, R01AD, R03BA, S), exclusion of mifepristone (G03), exclusion of metyrapone (V04), missing combinations | corticosteroids + antiinflammatory/antirheumatic agents (M01BA) | corticosteroids + local anesthetics (H02B) | H02B | corticosteroids (A01, D07, H02, M01, G01, R, S, ...) |
| H03 | thyroid therapy | therapeutic, pharmacologic | exclusion of iodine preparations for the prevention of mineral deficiency (A12CX) | - | - | - | hormones (H03), mineral deficiency (A12), vitamins (A, B, V)  (Bianco and Taylor 2024; Lee and Pearce 2024) |
| H04 | pancreatic hormones | - | exclusion of diazoxide (C02DA, V03AH), exclusion of insulins (A10A) | - | - | - | hypoglycemia (C02, V03), insulins (A10)  (Röder et al. 2016) |
| H05 | calcium homeostasis | therapeutic, pharmacologic | exclusion of vitamin D (A11CC) | - | - | - | hypercalcemia (M05), calcifediol (A11), vitamins  (Ruppe 2011; Bkaily and Jaques 2023) |
| **J** | **antiinfectives for systemic use** | | | | | | |
| J01 | antibacterials for systemic use | chemic, miscellaneous | exclusion of antimycobacterials (J04, e.g. dapsone), exclusion of oral formulations with vancomycin (A07A), exclusion of oral formulations containing colistin (A07A), exclusion of oral formulations containing imidazole derivatives (P01), exclusion of pessaries (G01), missing combinations | antibacterials + tuberculostatics (J04AM), antibacterials + local anesthetics/vitamins (J01), streptomycin + antimycobacterials (J04AM) | antibacterials + antiparasitics (J01R), quinolones + urine acidifier (J01MB), nitrofurans + urine acidifier (J01XE), nitrofurantoin + phenazopyridine (J01XE), other antibacterials + urine acidifier (J01XX) | J01CR, J01EE, J01R | antimycobacterials (J04), intestinal antiinfectives (A07A), antiprotozoals (P01), gynecological antiinfectives (G01) |
| J02 | antimycotics for systemic use | therapeutic, pharmacologic, chemic, miscellaneous | dermatological indication excluded even if systemic use (D01B), exclusion of fumagillin (P01AX), exclusion of ketoconazole (H02CA), exclusion of vaginal formulations of triazole derivatives (G01) | - | - | - | antiinfectives (A01, A07), antifungals for dermatological use (D01), gynecological antiinfectives (G01), antiprotozoals (P01), anticorticosteroids (H02CA)  (Carmo et al. 2023; Hoenigl et al. 2024) |
| ***Table 2 (continued)*** | | | | | | | |
| ***ATC code*** | ***group title*** | ***mixed classification criteria, other findings*** | ***excluded relevant drugs*** | ***combinations placed outside group*** | ***combinations with other drugs included*** | ***separate category*** | ***key redundancy and overlap*** |
| J04 | antimyco-bacterials | - | exclusion of streptomycin (J01G), exclusion of thalidomide (L04AX) | - | antimycobacterials + streptomycin (J04AM), isoniazid + rifampicin/tuberculo-statics (J04AM), isoniazid + iron (J04AC), "other drugs for treatment of tuberculosis" + pyridoxine (J04AK), antimycobacterials + vitamins/antibacterials for systemic use from J01 (J04AM) | J04AM | antibacterials (J01), amino salicylic acid, antineoplastic and immunomodulating agents (L04)  (Chauhan et al. 2021; Fatima et al. 2021) |
| J05 | antivirals for systemic use | therapeutic, pharmacologic | exclusion of amantadine (N04), exclusion of vaginal ring with dapiravine (G01AX), exclusion of plain antivirals with cobicistat (V03AX), missing combinations | ribavirin + peginterferon alfa-2a/b (L03AB) | - | J05AR | parkinson drugs (N04), various (V03)  (Fletcher et al. 2021) |
| J06 | immune sera and immuno-globulins | - | exclusion of antiviral monoclonal antibody nirsevimab (J06B), missing combinations | specific immunoglobulins + vaccines (J07) | immunoglobulin + hyaluronidase (J06BA) | - | vaccines (J07) |
| J07 | vaccines | therapeutic, miscellaneous | - | - | - | J07C | immune-sera/-globulins (J06) |
| **L** | **antineoplastic and immunomodulating agents** | | | | | | |
| L01 | antineoplastic agents | pharmacologic, chemic, miscellaneous | exclusion of radiopharmaceuticals for cancer treatment (V10X), exclusion of trimetrexate (P01AX), exclusion of methotrexate for non-cancer indications and oral formulations (L04AX), exclusion of antivirals for topical use (D06BB), exclusion of colchicine (M04AC), exclusion of oral formulations of sirolimus (L04AH), exclusion of low strength CD20 inhibitors (L04AG), exclusion of bevacizumab for AMD (S01LA), missing combinations | antineoplastic agents + detoxifying agents (V03AF) | HER2 + EGFR inhibitors (L01EH), monoclonal antibodies + hyaluronidase (L01FY) | L01XY | corticosteroids (H02), cancer treatment, antiinfectives (P01AX), L04, multiple sclerosis (L04), antivirals (D06), sensory organs (S01), various (V)  (Guichard et al. 2017; Goodman et al. 2024 ; Olejnik et al. 2024) |
| L02 | endocrine therapy | chemic, miscellaneous | exclusion of antigrowth hormones (H01BC) | - | polyestradiol + local anesthetics (L02AA) | - | sex hormones (G03), progestogens (A15), gonadotropin releasing hormones (H01CA)  (Patel et al. 2023) |
| L03 | immuno-stimulants | pharmacologic, miscellaneous | exclusion of levamisole (P02CE) | - | peginterferon alfa-2b/a + ribavirin (L03AB) | - | anthelmintics (P02), antiinfectives (J)  (Mohi-ud-din et al. 2023) |
| L04 | immuno-suppressants | therapeutic, pharmacologic, miscellaneous | exclusion of corticosteroids, exclusion of dimethyl fumarate (N04AX), exclusion of parenteral formulations of cladribine (L01BB), exclusion of interleukin inhibitors used in asthma (R03DX), exclusion of dupilumab (D11AH), exclusion of high strength formulations of ofatumumab for CLL (L01FA), exclusion of parenteral and topical dermatological formulations of mTOR kinase inhibitors (L01EG), exclusion of parenteral formulations of methotrexate (L01BA) | - | JAK inhibitors + TYK2 inhibitors (L04AF) | - | antineoplastic agents (L01), corticosteroids (D07, H02, G01, S, A, C, M, N, R, S, L), dermatologicals (D11), dimethyl fumarate, nervous system (N)  (Hussain and Khan 2022; Möhlmann et al. 2024; Hosseini et al. 2019) |
| ***Table 2 (continued)*** | | | | | | | |
| ***ATC code*** | ***group title*** | ***mixed classification criteria, other findings*** | ***excluded relevant drugs*** | ***combinations placed outside group*** | ***combinations with other drugs included*** | ***separate category*** | ***key redundancy and overlap*** |
| **M** | **musculo-skeletal system** | | | | | | |
| M01 | anti-inflammatory and antirheumatic products | therapeutic, chemic | exclusion of corticosteroids (H02), exclusion of most salicylic acid preparations (N02BA), exclusion of celecoxib used in familiar adenomatous polyposis (L01XX), exclusion of chloroquine and hydroxychloroquine (P01BA), missing combinations | antiinflammatory/  antirheumatic drugs + paracetamol (N02BE), antiinflammatory/  antirheumatic drugs + antibacterials (J01), antiinflammatory/ antirheumatic drugs + opioids (N02AJ), naproxen + sumatriptan (N02CC), celecoxib + amlodipine (C08CA) | salicylates + corticosteriods (M01B), antiinflammatory/ antirheumatic drugs + muscle relaxants (M03B), antiinflammatory/ antirheumatic drugs + other drugs (M01BX) | M01BX | corticosteroids (H02), aminosalicylic acid (A07), folic acid (L01), immunosuppressants (L04), aminoquinolines (P01), muscle relaxants (M03), antiinfectives (J01), opioids (N02), analgesics (N02), migraine (N02), cardiovascular system (C08), malaria (P01)  (Ghlichloo and Gerriets 2023; Wudexi et al. 2021; Okpala et al. 2025; Boechat et al. 2020) |
| M02 | topical products for joint and muscular pain | therapeutic, pharmacologic, chemic, miscellaneous | exclusion of diclofenac 3% hyaluronic acid gel (D11AX), exclusion of capsaicin for neuropathic pain (N01BX), exclusion of menthol (D04) | - | salicylic acid derivatives + other drugs (M02AC), salicylic acid derivatives + other non-steroidal antiinflammatory derivatives (M02AA) | - | dermatologics (D11, D04), anesthetics (N01)  (Vinyes et al. 2023) |
| M03 | muscle relaxants | - | exclusion of sugammadex as an antidot (V03AB) | - | muscle relaxants + analgesics/ corticosteroids (M03B), ethers + paracetamol (M03BC) | - | urologicals (G04), anesthetics (N01), antidotes (V03), analgesics (N02), corticosteroids (H02), COX inhibitors (M01)  (Beebe et al. 2005) |
| M04 | antigout preparations | pharmacologic, miscellaneous | exclusion of rasburicase (V03AF) | - | allupurinol + other antigout drugs (M04AA) | - | hyperuricemia (V03), colchicine (L01, M03)  (Sattui and Gaffo 2016; McKenzie et al. 2021; Zhang et al. 2022) |
| M05 | drugs for treatment of bone diseases | chemic, pharmacologic, miscellaneous | yes, highly incomplete, many drugs not listed and included in other groups, e.g. A11, A2, G03, H03BA | - | biphsophonates + calcium (M05BB) | M05BB | vitamins (A11), calcium and fluoride (A12), sex hormones (G03). calcitonins (H05)  (Tu et al. 2018; Chen et al. 2024a) |
| M09 | other drugs for disorders of the musculo-skeletal system | miscellaneous | catch-all group for M | chinine in combinations for cold conditions (R05X) | quinine + psycholeptics (M09AA) | - | quinine (P01), respiratory diseases (R05), hyaluronic acid (S01), anti-inflammatory drugs (M01), cardiovascular system (C) |
| ***Table 2 (continued)*** | | | | | | | |
| ***ATC code*** | ***group title*** | ***mixed classification criteria, other findings*** | ***excluded relevant drugs*** | ***combinations placed outside group*** | ***combinations with other drugs included*** | ***separate category*** | ***key redundancy and overlap*** |
| **N** | **nervous system** | | | | | | |
| N01 | anesthetics | - | exclusion of benzodiazepines (N05), exclusion of transdermal, nasal and sublingual formulations of fentanyl (N02AB), exclusion of nasal administration of esketamine (N06), exclusion of dermatological, ophthalmological use, throat preparations (D04AB, S01HA, R02AD), exclusion of lidocaine injections used as antiarrhythmics (C01BB), missing combinations | anesthetics + antihemorrhoidals (C05AD), anesthetics + stomatologicals (A01AD), anesthetics + corticosteroids for local oral treatment (A01AC) | barbiturates + other drugs (N01AG), anesthetics + epinephrine (N01B) | N01AG | psycholeptics (N05), muscle relaxants (M03A), barbiturates (N05C), MOR agonists (N02), psychoanaleptics (N06), dermatology (D04), vasoprotectives (C05), local oral treatment (A01), throat preparations (R02), sensory organs (S), local dental use (A01), antiarrhythmic drugs (C01), peripheral neuropathic pain (N01, M02)  (Becker and Reed 2012; Vinyes et al. 2023; Kim et al. 2022) |
| N02 | analgesics | therapeutic, pharmacologic, miscellaneous | exclusion of ibuprofen (M01A), exclusion of analgesics for specific indications (A03D/E, M01, M02A, M03), exclusion of lidocaine for postherpetic pain (N01BB), exclusion of sufentanil (N01AH), exclusion of plain codeine (R05D), exclusion of parenteral formulations of fentanyl (N01AH), exclusion of methadone, levomethadone, levacetylmethadol and high strengths of buprenorphine (N07BC), exclusion of salicylic acid for antithrombotic purpose (B01AC), exclusion of tolfenamic acid (M01AG), exclusion of botulinum toxin (M03AX) | salicylic acid + corticosteroids (M01B), fentanyl + bupivacaine (N01AH), dextropropoxyphene + muscle relaxant (M03B), sodium salicylate + methenamine (G04BX), paracetamol + orphenadrine (M03BC), indomethacin + prochlorperazine + caffeine (M01AB), dihydroergotamine + etilefrine (C01CA) | analgesics + psycholeptics (N02), MOR agonists + antispasmodics (N02AG), analgesics + codeine (N02AJ), MOR agonists + caffeine/histamine receptor antagonist/anticholinergic drugs (N02AA), salicylic acid + antiemetics (N02BA) | N02AG, N02AJ | musculo-skeletal system (M01, M02, M03), psycholeptics (N05), functional gastrointestinal disorders (A03), anesthetics (N01), antispasmodics (A03), antipropulsives (A07), respiratory disorders (R05), addictive disorders (N07), antithrombotic drugs (B01AC), urologicals (G04), anticonvulsants (N03), antivertigo drugs (N07), histamine receptor antagonist (R06A), ergot alkaloids (G02, G04), corticosteroids  (Queremel Milani et al. 2023) |
| N03 | antiepileptics | chemic, miscellaneous | exclusion of barbiturates used as hypnotics/sedatives (N05C), exclusion of gabapentin and pregabalin (N02BF) | - | barbiturates + phenytoin (N03AB) | - | hypnotics/sedatives/anxiolytics (N05)  (Nadkarni and Devinsky 2005) |
| N04 | anti-parkinson drugs | pharmacologic, miscellaneous | exclusion of orphenadrine citrate (M03), exclusion of low strength bromocriptine, cabergoline, lisuride (G02BC, G02CB, N02CA) | - | dopaminergic drugs + decarboxylase inhibitors (N02BA), levodopa + decarboxylase inhibitor + COMT inhibitor (N04BA) | - | muscle relaxants (M03), prolactine inhibitors (G02), analgesics (N02CA)  (Sivanandy et al. 2021; Kispotta et al. 2024) |
| N05 | psycholeptics | - | exclusion of reserpine (C02), exclusion of clonazepam (N03) | mGPCR antagonists + NE/5-HT enhancers (N06C), anxiolytics/psycholeptics/hypnotics + other drugs (A03, N02, N06, C02, C03, M03, R03, ...) | - | N05CB, N05CX | antihypertensives (C02), propulsives (A03), NE/5-HT enhancers (N06), analgesics (N02), antiepileptics (N03), anesthetics (N01)  (Seidel et al. 2013; Carnovale et al. 2023) |
| ***Table 2 (continued)*** | | | | | | | |
| ***ATC code*** | ***group title*** | ***mixed classification criteria, other findings*** | ***excluded relevant drugs*** | ***combinations placed outside group*** | ***combinations with other drugs included*** | ***separate category*** | ***key redundancy and overlap*** |
| N06 | psychoanaleptics | therapeutic, pharmacologic | exclusion of lithium (N05AN), exclusion of esketamine injections (N01), exclusion of clonidine and guanfacine (C02AC) | caffeine + respiratory stimulants (R07AB) | NE/5-HT enhancers + psycholeptics (N06C) | N06C | psycholeptics (N05), anesthetics (N01), antihypertensives (C02), anti-obesity drugs (A08), respiratory system (R07)  (Vedrenne-Gutiérrez et al. 2024; Smith Breault et al. 2025) |
| N07 | other nervous system drugs | therapeutic, pharmacologic, miscellaneous; catch-all group for N | exclusion of bupropion (N06A) | - | - | - | glaucoma therapy (S01), detoxification (V03), NE/5-HT enhancers (N06A), analgesics (N02), antivertigo drugs (A04, C04, N02, N05, R06), immunosuppressants (L04) |
| **P** | **antiparasitic products, insecticides and repellents** | | | | | | |
| P01 | antiprotozoals | limited number of listed parasites | exclusion of chlorquinaldol and clioquinol for dermatological use (D08AH), exclusion of nitroimidazoles for vaginal administration, parenteral formulation and treatment of *H. pylori* (G01AF, J01XD, A02BD), exclusion of hydroquinone (M09) | antiparasitics + antibacterials (J01R), quinine + psycholeptics (M09), qunine combinations for cold condtitions (R05X) | glycobiarsol + clioquinol (P01AA) | - | dermatologics (D08), gynecologicals (G01), antiinfectives (J), alimentary tract and metabolism (A02), biguanides (A10, D08), musculo-skeletal system (M09), respiratory diseases (R05)  (Wozel and Blasum 2014; Sawyer et al. 1976; Sweeney et al. 2003; Martínez-Girón et al. 2008) |
| P02 | anthelmintics | limited number of listed worm-types and therapeutics | - | - | - | - | antiinfectives (J) |
| P03 | ecto-parasiticides, incl. scabicides, insecticides and repellents |  | exclusion of crotamiton (D04AX), exclusion of dimeticone used as an antiflatulent (A03AX) | - | benzyl benzoate + sulfur (P03AA) | - | antipruritics (D04), antiflatulents (A03) |
| ***Table 2 (continued)*** | | | | | | | |
| ***ATC code*** | ***group title*** | ***mixed classification criteria, other findings*** | ***excluded relevant drugs*** | ***combinations placed outside group*** | ***combinations with other drugs included*** | ***separate category*** | ***key redundancy and overlap*** |
| **R** | **respiratory system** | | | | | | |
| R01 | nasal preparations | therapeutic, chemic, miscellaneous | exclusion of varenicline nasal spray (S01XA), exclusion of nasal preparations for eye, ear and nose (S03), exclusion of plain histamine receptor antagonists (R06), exclusion of cold preparations with therapeutic levels of analgesics/anti-inflammatory drugs (N02, M01) | tetracaine + oxymetazoline (N01BA) | muscarinic receptor antagonists + antibacterials/histamine receptor antagonist/ipratropium bromide (R01AB), corticosteriods + antiinfectives/ muscarinic receptor antagonists/histamine receptor antagonist/other drugs (R01AD), nasal decongestants + histamine receptor antagonist (R06), antiallergics + corticosteroids (R01AD) | R01AB | antiinfectives (J), sensory organs (S), respiratory system (R01, R06), anesthetics (N01), corticosteriods, analgesics (N02), antiinflammatory/antirheumatic drugs (M01)  (Safarov et al. 2024) |
| R02 | throat preparations | therapeutic, pharmacologic, miscellaneous | exclusion of drugs used in gingivitis, stomatitis (A01), exclusion of dental anaesthetics (N01B) | - | cetylpyridinum + lysozyme (R02AA), antiseptics + antibacterials (R02AB), anesthetics + antiseptics/ antibacterials (R02AA/B) | - | stomatological preparations (A01), antiinfectives (J, A01), anesthetics (R02)  (van der Sandt and Ramoleta 2016) |
| R03 | drugs for obstructive airway diseases | therapeutic, pharmacologic, miscellaneous | exclusion of ephedrine injections (C01) | - | xanthines + adrenergics (R03DB), xanthines + other drugs excl. adrenergics (R02DA), leukotriene receptor antagonists + histamine receptor antagonist (R03DC) | R03AH, R03AL, R03CK | corticosteroids (H2), anticholinergics (A03, N04, S01), hypotension (C01), respiratory stimulants (R07), rhinitis (R01), theophylline, histamine receptor antagonist (R06A)  (Linton et al. 2023; Cazzola et al. 2012 ; Restrepo 2007 ; Singh et al. 2025) |
| R05 | cough and cold preparations | therapeutic, pharmacologic, miscellaneous | exclusion of combinations when therapeutic levels of other drugs are included, exclusion of many application forms (R01, R02, R03D) | cold preparations with antiinfectives/analgesics (J, N02, M01), expectorants + adrenergics (R03C), mucolytics + antiinflammatories (M01) | expectorants + histamine receptor antagonist (R05C), mucolytics + xanthines (R03DA) | R05F, R05X | antiinfectives (J), analgesics (N02), anti-inflammatory drugs (M01), R, codeine  (Woo 2008; Kim et al. 2015) |
| ***Table 2 (continued)*** | | | | | | | |
| ***ATC code*** | ***group title*** | ***mixed classification criteria, other findings*** | ***excluded relevant drugs*** | ***combinations placed outside group*** | ***combinations with other drugs included*** | ***separate category*** | ***key redundancy and overlap*** |
| R06 | antihistamines for systemic use | chemic, miscellaneous; catch-all group for histamine receptor antagonists not classified elsewhere | exclusion of cinnarizine and flunarizine (N07C) | histamine receptor antagonist + analgesics (N02), histamine receptor antagonist + xanthines (R03DA), histamine receptor antagonist + leukotriene receptor antagonists (R03), histamine receptor antagonist + expectorants (R05C), histamine receptor antagonist + nasal decongestants (R01B), histamine receptor antagonist + cough supressants (R05D), aminoalkyl ethers + codeine (N02AA), cinnarizine + dimenhydrinate (N07CA) | - | R06AK | antiemetics (A04), antivertigo drugs (N07C), respiratory system (R), allergen extracts (V01), anti-inflammatory drugs (M01)  (Schaefer et al. 2024; Hunter et al. 2022; Canonica and Blaiss 2011) |
| R07 | other respiratory system products | therapeutic, pharmacologic, miscellaneous; catch-all group for R | exclusion of caffeine (N06B), exclusion of centrally acting respiratory stimulants (R03D) | - | - | - | psychostimulants (N06), nitric oxide (V03), R01-R06 |
| **S** | **sensory organs** | | | | | | |
| S01 | ophthalmo-logicals | therapeutic, pharmacologic, miscellaneous; catch-all group for any preparations used in the eye | exclusion of hyaluronic acid injection for intra-articular administration (M09A), exclusion of mitomycin (L01) | S03 | antibacterials + other drugs (S01AA), muscarinic receptor agonists/acetylcholine esterase inhibitors + epinephrine (S01EB), muscarinic receptor agonists/acetylcholine esterase inhibitors +beta-adrenergic receptor antagonists (S01ED), low strength phenylepinephrine + other drugs (S01GA) | S01BB, S01CB | antiinfectives, corticosteroids, analgesics, anesthetics, muscarinic receptor antagonists, beta-adrenergic receptor antagonists, prostaglandines, antiallergics, diagnostic drugs, musculo-skeletal system (M09), L  (Ahmed et al. 2023) |
| ***Table 2 (continued)*** | | | | | | | |
| ***ATC code*** | ***group title*** | ***mixed classification criteria, other findings*** | ***excluded relevant drugs*** | ***combinations placed outside group*** | ***combinations with other drugs included*** | ***separate category*** | ***key redundancy and overlap*** |
| S02 | otologicals | therapeutic, pharmacologic, miscellaneous; catch-all group for any preparations used in the ear | exclusion of corticosteroids | S03 |  | S02C | antiinfectives, corticosteroids, analgesics, anesthetics  (Kaushik et al. 2011) |
| S03 | ophthalmological and otological preparations | therapeutic, pharmacologic, miscellaneous | - | - | - | S03C | antiinfectives, corticosteroids, analgesics, anesthetics, ... |
| **V** | **various** | | | | | | |
| V01 | allergens | exclusion of diagnostic use (V04CL) | - | - | - | - | diagnostic allergens (V04) |
| V03 | all other therapeutic products | therapeutic, pharmacologic, chemic, miscellaneous | catch-all group for drugs not classifiable somewhere else | - | - | - | antidotes (B03, A07, A03, M01, A05, N07, N02), hyperphosphatemia (A12), detoxifiers in antineoplastic treatment (R05, M04), hypercalcemia (M05, H05), hypoglycemia (C02), tissue adhesives (B02), medical gases (R07) |
| V04 | diagnostic agents | catch-all group for all main groups | exclusion of aminolaevulinic acid (L01XD), exclusion of parenteral nutrition (B05BA) | - | - | - | V01, L01, nutrition (B05) |
| V06 | general nutrients | miscellaneous category; catch-all group for all main groups | exclusion of parenteral nutrition (B05BA) | - | - | - | antiobesity drugs (A08), vitamins, mineral supplements |
| V07 | all other non-therapeutic products | catch-all group for all main groups | exclusion of several plasters (D02AD, D09) | - | - | - | plasters (D02, D09), A, J, G, D |
| V08 | contrast media | catch-all group for all main groups | - | - | - | - | radiopharmaceuticals (V10) |
| V09 | diagnostic radio-pharmaceuticals | catch-all group for all main groups | - | - | - | - | radiopharmaceuticals (V09) |
| V10 | therapeutic radio-pharmaceuticals | catch-all group for all main groups | - | - | - | - | - |
| V20 | surgical dressings | catch-all group for all main groups | - | - | - | - | - |

***Table S2:*** *Key differences between the international ATC system and the modified German ATC system.*

| **Aspect** | **Difference German vs. international ATC system (Günther et al. 2025; WHO 2024)** |
| --- | --- |
| additional ATC groups | addition of phytotherapeutic, anthroposophic, and homeopathic groups in P and H (levels 2–4) |
| differing DDDs | differing DDDs for drugs/preparations, or supplemented DDDs for drugs without a DDD in the international classification |
| additional drugs | additional phytotherapeutic, anthroposophic, homeopathic, cell/gene therapies, and innovative drugs |
| additional ATC groups for combination preparations | additional ATC codes for combination preparations, assigned at a separate 5th level; relevant for the German pharmaceutical market but of low international relevance |
| allocation | different allocation of drugs when the main indication differs in Germany from the international system; in international comparisons, drugs are assigned according to the original classification (Table 5) |

***Table S3:*** *Differently allocated drugs in the German versus the international ATC system (Günther et al. 2025; WHO 2024), illustrating the ambiguity and multiple options for assigning preparations to ATC groups. Drugs and substances are sorted descending by the ATC code of the international ATC system.*

| **Drug/ substance** | **Indication** | **German ATC system** | **International ATC system** |
| --- | --- | --- | --- |
| magnesium carbonate | mineral deficiency | A12CC | A02AA |
| moxaverin, papaverin | peripheral circulatory disorders | C04AX | A03AD |
| peppermint leaves | not specified | A03AP | A03AX |
| silymarin, phsopholipids | not specified | A05BP | A03BA |
| thioctic acid | diabetic polyneuropathy | N07XB | A05BA |
| decaying tree bark | diagnosis purpose | V04CZ | A06AB |
| sulfasalazine | rheumatoid arthritis | M01CX | A07EC |
| ceratonia | not specified | A07XP | A07XA |
| bromelain | not specified | M09AP | A09AB |
| liraglutide, semaglutide | not specified | A08AX | A10B |
| guar flour | not specified | A10XP | A10BX |
| dexpanthenol, high strength, parenteral | postoperative intestinal atony | A03FA | A11HA |
| calcium | pathologic serum phosphate level | V03AE | A12AA |
| zinc | acne | D10BX | A12CB |
| ademetionine | inflammatory degenerative joint diseases | M09AX | A16AA |
| dipyridamole | coronary insufficiency | C01DX | B01AC |
| selexipag | pulmonary artery hypertension | C02KX | B01AC |
| sildenafil, tadalafil, iloprost, selexipag | pulmonary artery hypertension | C02KX | B01AC, G04BE |
| enzyme-containing artery-venous shunts | not specified | B01AY | B01AD |
| natriumpentosanpolysulfate | systemic thrombolysis | B01AX, C05B | B05B |
| glycosides, phytotherapeutic | not specified | C01AP, C01AA, C01AC | C01AA, C01AC |
| convallariaglycosides | not specified | C01AP | C01AX |
| mexiletin | myotonia | M09AX | C01BB |
| dehydroergotamine | hypotonia | C06A | C01C |
| alprostadil, iloprost | peripheral circulatory disorders | C04AG | C01EA, G04BE |
| rauwolfia | not specified | C02AP | C02AA |
| reserpine | psychosis | N05AX | C02AA |
| guanfacine | ADHD | N06BA | C02AC |
| tolvaptan | Autosomal-dominant polycystic kidney disease | G04BX | C03XA |
| vasodilators | dementia | N06DX | C04A |
| horse chestnut seeds | not specified | C05CP | C05CX |
| ***Table S3 (continued)*** | | | |
| ***drug/ substance*** | ***indication*** | ***German ATC system*** | ***International ATC system*** |
| bromelain, birch bark | not specified | D03AP | D03AX, D03BA |
| sinecatechins | not specified | D05BP | D06BB |
| crataegus glycosides | not specified | C01EP | E01EB |
| phytotherapeutics, homeopathics | gynecologic conditions | G02CH, G02CP | G02C |
| phytotherapeutics | urologicals | G02CH, G02CP | G02CX |
| chlorotrianisene | prostate cancer | L02AA | G03CA |
| terazosin | essential hypertension | C02CA | G04CA |
| phytotherapeutics | prostate related conditions | G04CP | G04CX |
| gonadotropine releasing hormone analogues | not specified | H01CA, L02AE | L02AE |
| dimethyl fumarate | plaque psoriasis | D05BX | L04AX |
| methotrexate | rheumatoid arthritis | M01CX | L04AX |
| bufexamac, dermatologics | eczema, neurodermitis | D04AX | M01AB, M02AB |
| phytotherapeutics | joint and muscle pain | M02AP | M02AX |
| buprenorphin | substitution in addictive disorders | N07BC | N02AE, N07BC |
| etilefrin + dihydroergotamin (combination) | hypotonia | C06AA | N02CA |
| barbiturates | hypnotics, sedatives | N05CA | N03 |
| amantadine | viral infections | J05AC | N04BB |
| lavender oil | not specified | N05BP | N05BX |
| midazolam | status epilepticus | N03AE | N05CD |
| idebenone | leber`s hederitary optic neurpathy | S01XA | N06BX |
| cinnarizine | dementia | N06DX | N07CA |
| quinine, monopreparations | nocturnal leg cramps | M09AA | P01BC |
| pyrethrum | not specified | P03AP | P03AC |
| phytotherapeutics | expectorants | R05CP, R05DP | R05CA |
| histamine receptor antagonists | antiemetic effect | A04AB | R06 |
| cyproheptadine, pizotifen | appetite stimulant | A15AA | R06AX, N03CX |
| organ-conserving solutions | organ transplantation | B05CB | V07AB |
